# Bridging Autism Spectrum Disorders and Schizophrenia through inflammation and biomarkers - pre-clinical and clinical investigations

**DOI:** 10.1186/s12974-017-0938-y

**Published:** 2017-09-04

**Authors:** Joana Prata, Susana G. Santos, Maria Inês Almeida, Rui Coelho, Mário A. Barbosa

**Affiliations:** 1 FMUP-Faculty of Medicine, University of Porto, Al. Prof. Hernâni Monteiro, 4200-319 Porto, Portugal; 2 i3S-Instituto de Investigação e Inovação em Saúde, University of Porto, Rua Alfredo Allen 208, 4200-135 Porto, Portugal; 3 INEB-Instituto de Engenharia Biomédica, University of Porto, Rua Alfredo Allen 208, 4200-135 Porto, Portugal; 4 ICBAS-Instituto de Ciências Biomédicas Abel Salazar, University of Porto, Rua de Jorge Viterbo Ferreira 228, 4050-313 Porto, Portugal

**Keywords:** Autism spectrum disorders, Schizophrenia, Inflammation, Animal model, Clinical research, Biomarker, Anti-psychotics, Immune cells, microRNA, Microglia

## Abstract

In recent years, evidence supporting a link between inflammation and neuropsychiatric disorders has been mounting. Autism spectrum disorders (ASD) and schizophrenia share some clinical similarities which we hypothesize might reflect the same biological basis, namely, in terms of inflammation. However, the diagnosis of ASD and schizophrenia relies solely on clinical symptoms, and to date, there is no clinically useful biomarker to diagnose or monitor the course of such illnesses.

The focus of this review is the central role that inflammation plays in ASD and schizophrenia. It spans from pre-clinical animal models to clinical research and excludes in vitro studies. Four major areas are covered: (1) microglia, the inflammatory brain resident myeloid cells, (2) biomarkers, including circulating cytokines, oxidative stress markers, and microRNA players, known to influence cellular processes at brain and immune levels, (3) effect of anti-psychotics on biomarkers and other predictors of response, and (4) impact of gender on response to immune activation, biomarkers, and response to anti-psychotic treatments.

## Background

Schizophrenia and autism spectrum disorders (ASD) are chronic and debilitating psychiatric disorders with devastating effects for patients and families. While ASD is a neurodevelopmental disorder of childhood, schizophrenia is diagnosed later on and affects mostly young adults. Also, the incidence of schizophrenia has remained stable across time, while the incidence of ASD has markedly increased over the last few decades. Such an increase cannot be fully explained by better diagnostic criteria or by greater medical awareness [1]. Both conditions are highly heritable, with 25–33% genetic contribution to schizophrenia and 49% to ASD [2]. Both disorders share some genetic influences with impairments in social communication, but display distinct developmental profiles of their genetic links, which is in agreement with their onset and clinical symptoms [2]. A previous study analyzing the shared genetic etiology between different psychiatric conditions found that overlap between schizophrenia an ASD was small, when compared with its overlap with adult onset psychiatric disorders [3]. Nonetheless, in very recent studies copy number variations, particularly in the 22q11, were found to be common to both conditions, indicating some genetic overlap between ASD and schizophrenia [4, 5]. Moreover, recent evidence suggests that alterations in gene expression regulation, synaptic architecture and activity, and immunity, are main cellular mechanisms contributing to both conditions [5]. Also, genetic risk factors need to be analyzed in the context of the interdependent interactions between genetic and environmental factors that play key roles in disease pathogenesis [6]. In fact, increasing evidence suggests that complex gene-environment interactions are at play in both schizophrenia and ASD [6].

**Fig. 1:**
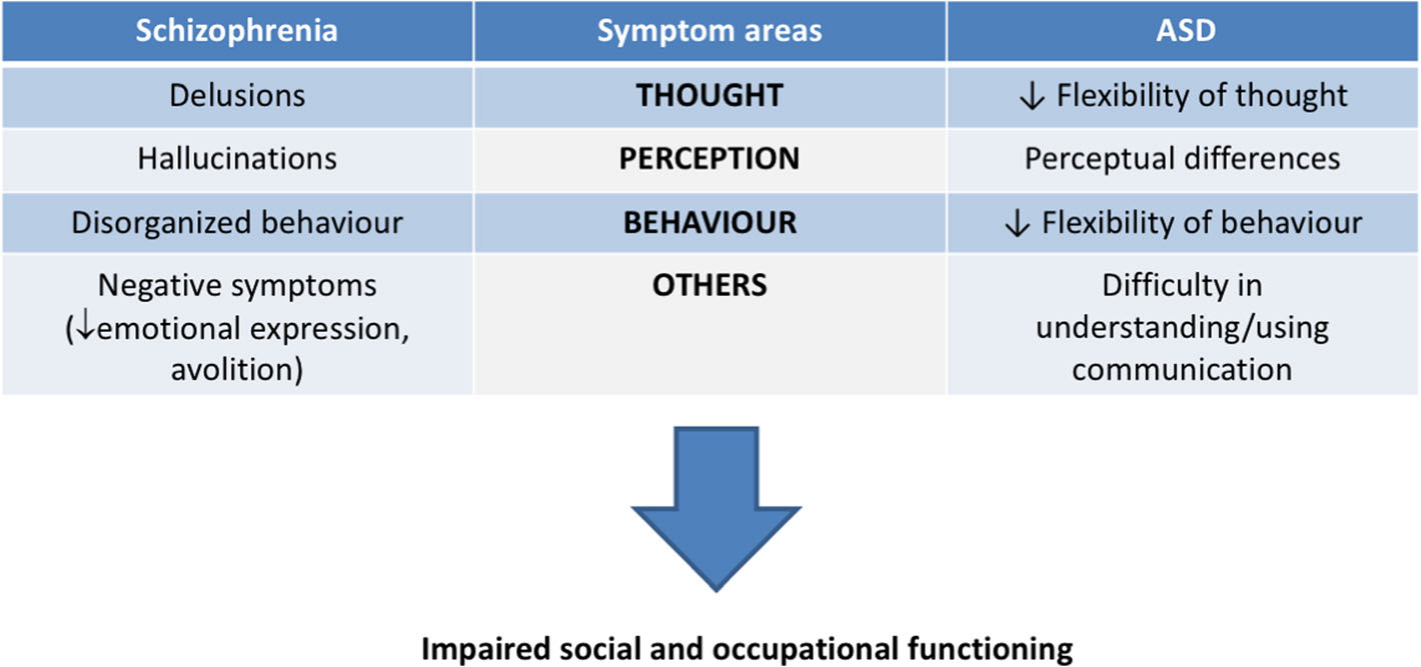
Clinical similarities between schizophrenia and ASD. The major symptom areas for the two conditions and their common functional outcome

Clinically, both disorders share some similarities regarding clinical symptoms, and this might reflect a similar biological basis, namely, in terms of inflammatory processes. The reasons underlying the clinical detection of ASD in early childhood, while schizophrenia is only diagnosed later on, remain largely to be explained. The largest study of childhood onset schizophrenia (diagnosis of schizophrenia before age 13) found a 28% comorbidity with autism or ASD [7], and cohort studies on the antecedents of schizophrenia report the existence of subtle developmental delays long before the onset of psychosis [7]. Differences in neuroinflammatory processes, in individual ability to mount appropriate immune responses, and the role of environmental factors on the brain’s maturational processes might all contribute to the differences in age onset, as further reviewed in [8]. In schizophrenia, the presence of delusions (thought disorder), disorganized speech (and/or behavior), and perceptual differences such as hallucinations, contributes to impaired social and occupational functioning as well as difficulties in interpersonal relationships. These symptoms are sometimes referred to as positive symptoms, that is, symptoms which are not present in the healthy individual (and have been added with the illness). Negative symptoms refer to symptoms which are lacking but used to be present in the healthy individual and include diminished emotional expression and avolition (decrease in the motivation to initiate and perform self-directed purposeful activities). In ASD, symptoms follow a continuum from mild to more severe. Typically, children have decreased flexibility of thought and behavior (repetitive patterns of behavior, interests, and activities), perceptual differences (hyper or hyporeactivity to sensory stimuli, fragmented and distorted perception, delayed perception, and sensory overload), difficulty in effectively using communication and social interaction. In children with communication deficits, it is virtually impossible to determine the presence of delusions and/or hallucinations. Fig. 1 summarizes the major symptom areas for ASD and schizophrenia. Both disorders share the same core symptom areas, which despite disorder-specific differences within each area, all contribute to impaired social and occupational functioning.

The diagnosis of both schizophrenia and ASD relies solely on clinical symptoms and, to date, there is no clinically useful biomarker to determine diagnosis, course of illness, or response to pharmacological treatment. Current available pharmacological treatment for schizophrenia consists on the use of anti-psychotic medication, which has considerable adverse side effects. As a result, patient non-compliance, non-responsive, or partially responsive to treatment are common. This is particularly important as it is well established that the prognosis of psychotic disorders is directly impacted by the duration of untreated illness and adherence to treatment [9]. In the case of ASD, there is no current available treatment for the core symptoms of the disorder and, so far, pharmacological interventions (anti-psychotics, stimulants, and anti-depressives) aim to attenuate non-core symptoms. Further, the long-term consequence of their use in children remains largely unknown.

Over the last decade studies have focused on the pathogenesis of schizophrenia and ASD and many potential pathways have been identified, with studies reporting differences between patients and controls on a variety of biomarkers. In patients with schizophrenia, a vast array of altered molecular profiles in levels of immune-inflammatory markers, growth factors, hormones, elements of oxidative stress pathways, and of protein and lipid metabolism pathways, have been reported and are reviewed elsewhere [10–12]. In ASD, similar physiological pathways and mechanisms appear to be altered, namely, the immune system, inflammation, oxidative stress, free fatty acid metabolism, mitochondrial function, and the balance between excitatory and inhibitory pathways. A wide range of potential biomarkers for ASD has also been identified in each physiological pathway, and have been subject of other reviews [13, 14]. Search for clinical biomarkers to predict schizophrenia and ASD diagnosis and clinical outcomes has been widely expanded in the last decade, which could support patient stratification, early detection of the disease, and clinical decision-making [15, 16]. Both schizophrenia and ASD have been associated with chronic and low-grade inflammatory state [17, 18]; therefore, it is not surprising that a considerable number of pro-inflammatory biomarkers, including cytokines such as interleukin (IL)-6, tumor necrosis factor (TNF)-α, IL-1β, chemokine (C-X-C motif) ligand 8 (CXCL8, also known as IL-8), interferon (INF)-γ, among others [17–19], have been identified. However, these protein biomarkers can so far only partially determine the disease signature in the clinical setting. Hence, novel biomarkers are being investigated.

**Fig. 2:**
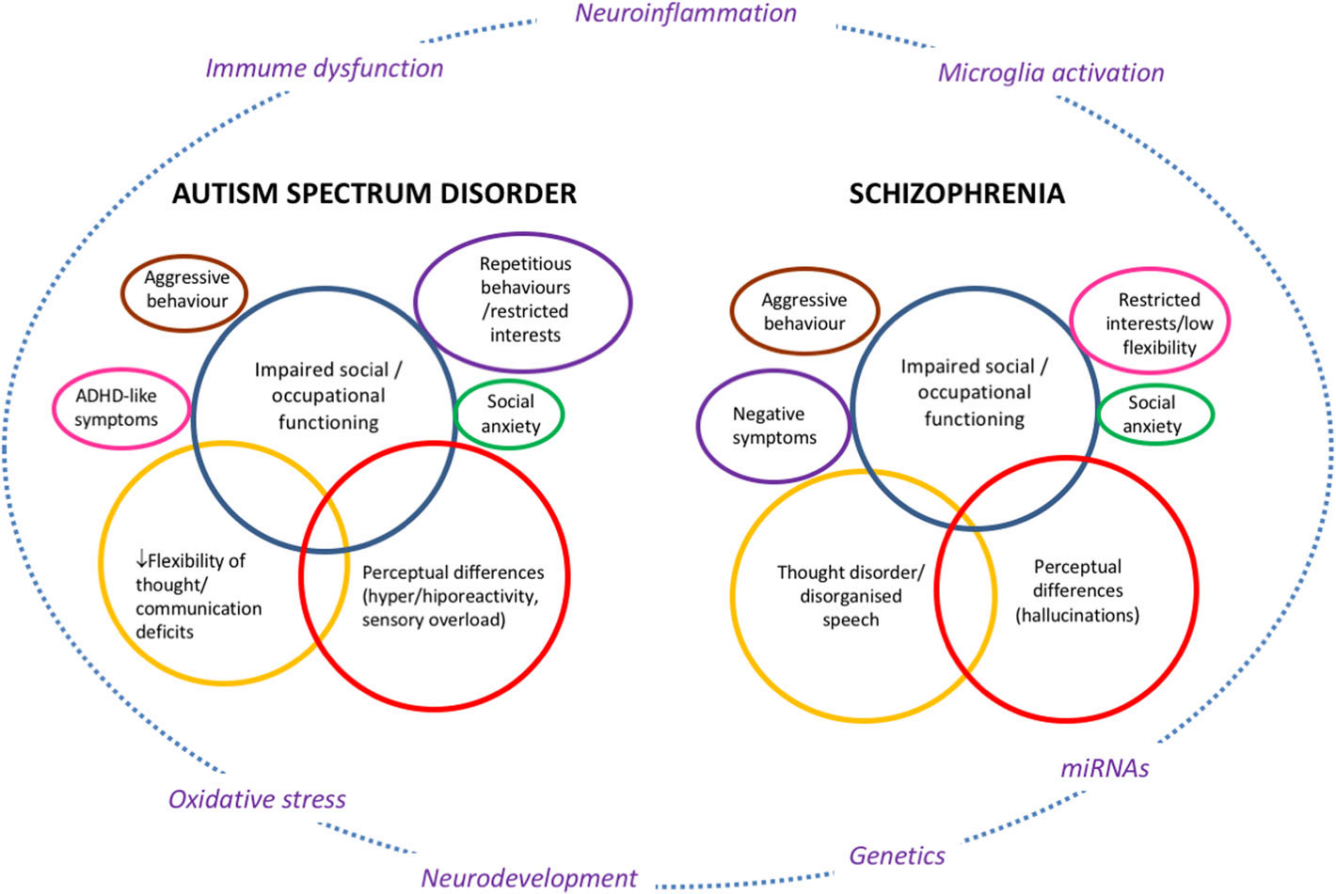
An integrative model of autism spectrum disorder and schizophrenia. In this hypothetical model of the interactions between ASD and schizophrenia, the center circles with the same color and their intersections illustrate the similarities between ASD and schizophrenia, in terms of clinical symptom areas, while the underlying biological processes are represented in the outer circle

In this review, we hypothesize that similarities between ASD and schizophrenia are linked to a shared biological basis, namely, inflammation. Figure 2 illustrates our hypothetical integrative model of schizophrenia and ASD. We propose that similarities in the major clinical symptom areas (inner circles) might share common underlying biological processes (depicted by the outer circle). Here, we also aim to integrate current knowledge from animal studies, explore the importance of microglia, and discuss how biomarkers relate to clinical features, course of illness and to anti-psychotic treatment in patients with schizophrenia and ASD. The impact of gender in both conditions is also discussed.

## Pre-clinical research: inflammation in animal models of ASD and schizophrenia

This review focuses on a selection of animal models for ASD and schizophrenia that represent different contributing factors for disease development, and where the link with inflammation has been reported (Table 1). Although an animal model will not likely be able to display all behavioral features of the human condition, some traits can be evaluated that have correspondence in humans. Determining the degree of face validity in animal models is of particular importance in neuropsychiatric disorders. So, several tests have been developed to probe aspects of social interaction, evaluating the presence of stereotypy and repetitive behaviors, and investigating despair behavior. Examples of social interactions tests are the multicage set ups that allow the test animal to choose to contact or not with new animal(s), as reviewed and illustrated in [20, 21]. Also, mice can reveal spontaneous motor stereotypies, such as high levels of circling, flipping, vertical jumping, and sniffing one location, and repetitive behaviors, like unusually long bouts of self-grooming, digging, and burying foreign objects [20, 22]. New technologies have improved animal monitoring, such as the use of video recording equipment to investigate the presence of stereotypical behavior [23], or the use of magnetic resonance imaging (MRI), allowing in vivo neuroanatomical and functional studies of the brain [22]. These tests, in the context of parallelism between animals and humans that suffer from ASD, have been reviewed recently [20, 22].

**Table 1:** Inflammation in animal models of ASD and schizophrenia

Disease	Animal model	Trigger	Main features and outcomes	Inflammatory molecules, cells and processes	References
Genetic models					
ASD	BTBR mice	Crossing of inbred strain a^t^ (non-agouti; black and tan) and wild-type T (brachyury) mutations, with mice carrying tufted (Itpr3tf) allele	Avoid social interaction; increased repetitive behaviors; altered functional connectivity; loss of corpus callosum; formation of the cortical area and interhemispheric connectivity altered in an age- and region-specific manner; altered oxidative stress mechanisms	Higher brain-reactive IgG levels; increased microglia activation; increased pro-inflammatory cytokines (IL-1b, IL-18 and IL-33, IL-6, IL-12); decreased B cells; increased numbers of CD4 T cells; enhanced M1 macrophage polarization	Careaga et al. 2015; Fenlon et al. 2015; Heo et al. 2011; Hwang et al. 2015; Kim et al. 2016; Meyza et al. 2013; Onore et al. 2013; Sforazzini et al. 2014; Shpyleva, Ivanovsky, 2014
ASD/Rett syndrome	Mecp2 mutant mice and non-human primates	Mecp2 gene mutation or duplication	Model of Rett syndrome. Repetitive locomotion, increased anxiety, reduced social interaction, and relatively weak cognitive phenotypes	Activation, followed by loss of microglia and some monocytes and macrophage populations (e.g., meningeal)	Cronk et al. 2015; Liu et al. 2016
Schizophrenia	DISC1 mutant mice	DISC1 gene mutation	Alteration in brain connectivity and function during development; behavioral and cognitive impairments; anatomical, cell biological, and circuitry deficits	Impaired GSK-3b signaling; higher IL-1b and IL-5 in fetal brain	Flores, Morales-Medina, 2016; Tomoda et al. 2016; Abazyan et al. 2010
Neurodevelopmental models					
ASD	Pre-natal stress/exposure models	e.g., administration of VPA during pregnancy	Decreased social interaction in adult males; reduced cognitive function	Increased basal glial activation; altered systemic inflammation; changes in immunity-related gene expression; increased intestinal inflammation	Lucchina and Depino, 2014; Huang et al. 2016; de Theije et al. 2014; Hill, 2015
ASD and schizophrenia	Maternal immune activation (MIA) in mice or primates	Maternal infection or immune stimulation (LPS, PolyI:C,)	Reduced PPI and ultrasonic vocalizations; decreased sociability and increased repetitive or stereotyped behavior; involvement of parvalbumin expressing interneurons in medial pre-frontal cortex; neurochemical and brain morphological abnormalities (enlarged ventricles; spatially localized deficit in Purkinje cells); decreased neurogenesis; impaired synaptic development	Maternal cytokine upregulation (IL-1β, IL-6, TNF-α IL-17, IL-10); fetal and neonatal increased cytokine levels in areas of the brain (frontal and cingulated cortex, and hippocampus) and in serum; increased cytokine and chemokine expression in the fetal brain; controversy on increased levels of microglial activation	Choi et al., 2016; Canetta et al. 2016; Garay, Hsiao, 2013; Smith et al. 2007; Juckel et al. 2011, Mattei et al. 2014, Van den Eynde et al. 2014; Pratt, Ni, 2013; (Giovanoli et al. 2015, Missault, Van den Eynde, 2014, Smolders et al. 2015; Coiro et al. 2015; Machado et al. 2015)
Schizophrenia	Neonatal ventral hippocampal lesion	Microinjection of Ibotenic acid in the ventral hippocampus at postnatal day 7	Abnormal behavioral phenotypes after puberty; Persistent astrogliosis and microglial activation; increase in metabotropic glutamate receptor type 5 (mGluR5); hippocampal neuronal loss	Persistent astrogliosis and microglial activation; Increased production of inflammatory mediators; Increased mGluR5 expression in astrocytes and microglia	Drouin-Ouellet et al. 2011; Hill, 2015
Combination models					
Schizophrenia	Combination of genetic and environmental factors (G × E)	MIA in the DISC1 mutant models	Developmental stage-specific deficits in social behavior, spatial working memory, and PPI; decreased volume of amygdala, hypothalamus, and periaqueductal gray matter; decreased 5-HT metabolism	Higher IL-6 in response to polyI:C in fetal brain in DiSC1 mutant mice; high IL-1β, reduced GSK-3β signaling and IL-5 in response to PolyI:C	Abazyan et al. 2010, Meyer, 2014; O’Leary, Desbonnet, 2014; Lipina et al. 2013
ASD and schizophrenia	Combination of 2 environmental factors (E × E)	MIA model combined with other post-natal stressors, like being reared by stressed mother, or pubertal stress exposure	Impaired working and spatial memory from pre-natal, but not postnatal maternal influence; combination of MIA with peri-pubertal stress led to neuropathological effects in the hippocampus GABAergic cell population	Transient neuroinflammation	Giovanoli, Weber, 2014; Richetto, Calabrese, 2013; Meyer, 2014

There are more than 70 reported models for ASD. In general, rodents are preferred and mice are the most used. However, other animals including non-human primates, are also employed to model these complex disorders. A recent study has analyzed different brain regions of 26 mouse models of ASD, using MRI-based neuroanatomical phenotyping [24]. Despite the heterogeneity of models, they can be clustered into different groups, based on abnormal alterations in particular areas of the brain, like the parieto-temporal lobe, cerebellar cortex, frontal lobe, hypothalamus, and the striatum [24]. These in vivo imaging studies in mouse models are invaluable in furthering knowledge on neuropsychiatric disorders in humans.

The first animal model developed for schizophrenia was based on the administration of amphetamines, as it was believed that schizophrenia was derived from hyperfunction of the dopaminergic (DAergic) neurotransmission, in the mesolimbic neuronal system. This was assumed because the DA D2-receptor antagonist was found to relieve the positive symptoms of schizophrenic patients, as described in a recent review [25]. Another pharmacological model was based on evidence indicating that there is hypofunction of the glutamatergic neuronal system in schizophrenia. The administration of phencyclidine (PCP), dizocilpine (MK-801), or ketamine induces the positive symptoms seen in patients with schizophrenia, via blocking of the *N*-methyl-D-aspartate (NMDA) receptor, which led to the NMDA-receptor antagonist model of schizophrenia.

### Modeling the genetic contribution to ASD and schizophrenia

Both ASD and schizophrenia etiology have important genetic components, with high heritability. However, multiple sites in the genome appear to be associated with the development of these and other mental disorders. Currently, most genetic animal disease models are rodents, particularly mice, and focus on highly penetrant rare mutations.

Some inbred mouse strains that are sometimes used as background for genetic mouse models display behavior abnormalities akin to human mental disorders, and can be used as models of psychiatric diseases. Many were characterized as part of the Mouse Phenome Project (MPP), which resulted in the mouse phenome database (http://phenome.jax.org), and two studies that followed it [21, 26].

The BTBR mice (also named BTBR T + tf/J) were obtained by crossing an inbred strain, which carried the a^t^ (nonagouti; black and tan) and wild type T (brachyury) mutations, with mice carrying the tufted (Itpr3tf) allele. These animals were characterized as part of the MPP and were found to have neuroanatomical abnormalities such as a hereditary loss of corpus callosum. Moy and colleagues characterized behavior abnormalities in the BTBR strain, confirming its face validity to ASD [21]. These animals show behaviors resembling human ASD core characteristics, such as avoiding social interaction and increasing repetitive behaviors [27]. BTBR mice have been used to study aging in ASD [28], with formation of the cortical area and interhemispheric connectivity being altered in an age- and region-specific manner in BTBR mice [29]. Also, studies combining MRI, histological and immunohistochemical analysis showed that BTBR mice have altered functional connectivity, recapitulating neuroimaging findings in human ASD [30]. Analyzing the cerebellum of BTBR mice showed important differences in expression of genes associated with DNA damage, chromatin organization, and cell death signaling [31]. Significant differences in enzymes related to oxidative damage to DNA, and modified DNA methylation, were found both in BTBR mice and in ASD human patients [31]. Oxidative stress-related biomarkers in humans, and the effect of anti-psychotics on these mediators, are discussed in Sections 4.1.2 and 4.2.2. A recent transcriptomic and proteomic analysis of the hippocampus of BTBR mice found modifications in genes previously associated with ASD in human patients, such as brain-derived neurotrophic factor (BDNF), SH3 and multiple ankyrin repeat domains 3 (Shank3), or extracellular signal-regulated kinase (ERK)1 [32].

Inflammatory mediators profile, including levels of immunoglobulins (Ig)G in the fetal brain of BTBR mice, have been characterized. BTBR mice had lower levels of glial fibrillary acidic protein (GFAP), and higher levels of brain-reactive immunoglobulin (IgG), except IgG1, than FVB mice [33]. This is in contrast with reports in humans showing increased GFAP levels in children with ASD [34]. The levels of brain-reactive IgG subtypes were recently investigated, with results indicating that levels of serum IgG1, IgG2b, and IgG3 in post-natal day 21 BTBR mice were higher than for FVB mice, regardless of sex [35]. Also, BTBR mice had increased activation of the microglia, higher levels of pro-inflammatory cytokines, particularly the IL-1 family (IL-1β, IL-18, and IL-33), and increased numbers of CD4 T cells [36]. Further studies showed also elevated IL-6 and IL-12, and a polarization tendency towards the pro-inflammatory M1 macrophage phenotype [37, 38]. Interestingly, as reviewed in the next section, microglial activation has also been reported in human autistic children, in a scenario of neuroinflammation, with increased levels of oxidative stress mediators and pro-inflammatory cytokines, such as IL-6, TNF-α, and IFN-γ [34]. Conversely, BTBR mice show decreased B cells, but increased antibody titters, while children with ASD display increased numbers of B cells [37].

The BTBR model has been used to study potential new therapeutic targets, such as (i) the peroxisome proliferator activated receptor—alfa (PPAR-a), which can be stimulated by the drug risperidone, decreasing repetitive behavior in BTBR animals [39], (ii) new forms of treatment for ASD, such as electroconvulsive therapy [40], and (iii) the effect of new drugs, like methyl-6-(phenylethynyl)-pyridine (MPEP) [41]. Also, environmental factors have been investigated with the BTBR model, such as (i) high-fat diet, which was found to exacerbate the cognitive rigidity and social deficits of the BTBR mouse [42], and (ii) organophosphate insecticides, which cause developmental neurotoxicity at subtoxic doses, showing a potential for aggravating the motor patterns of neonatal mice. In adulthood, it associated with altered pattern of investigation of a sexual partner, enhanced from the previously described for BTBR mice, and increased ultrasonic vocalization rate [43].

Transgenic animal models have been used to investigate the contribution of several genes found mutated in ASD and other developmental brain disorders. The most promising candidates for generating valuable ASD animal models have been recently reviewed in [44, 45]. A number of genes classified as transcriptional or epigenetic regulators have been implicated in ASD. An example is the methyl-CpG-binding protein 2 (MeCP2), which is associated with Rett syndrome, and has a crucial role in transcriptional regulation and miRNA processing. More than 10 distinct lines of MeCP2 mutant mice have been produced. Among them, the female heterozygous mice (MeCP2^246_/+^) are the model with best construct validity for Rett syndrome, as this disorder primarily affects females and is mostly lethal in males [45]. Cellular and molecular abnormalities have been identified in MeCP2 mutant mice that likely contribute to the ASD-like phenotypes, but the underlying mechanisms are not fully understood. Studies in this model suggest that distinct cellular entities, particularly astroglia and microglia, may have a substantial role in the neurobiology of Rett syndrome. In MeCP2 mice, there is an activation, followed by loss, of microglia and some monocyte/macrophage populations, during disease progression [46]. MeCP2 expression has been specifically and temporally targeted in mouse brain by using various Cre recombinase expressing lines. This approach allows to determine the contribution of specific brain regions, cell types, and developmental time periods for disease progression and symptoms. A recent study in non-human primates has shown that duplication of MeCP2 leads to autism-like behaviors such as repetitive locomotion, increased anxiety, reduced social interaction, and relatively weak cognitive phenotypes [47]. The authors generated an animal model where changes of MeCP2 only occur in brain tissues, demonstrating that non-human primates can be genetically engineered to study complex brain disorders [47]. Nonetheless, use of these animals has to be limited to absolutely essential experiments.

Meta-analysis have been performed to attempt to uncover the genes associated with schizophrenia, and many risk factor molecules, such as the NMDA receptor subunit 1 and 2A (NR1 and NR2A), disrupted in schizophrenia 1 (DISC1), neuregulin 1 (NRG1), dysbindin, and reelin have been proposed. Genetic models of schizophrenia have recently been reviewed [48].

DISC1 was initially identified as a gene disrupted by a translocation mutation that co-segregated with severe mental illnesses, such as schizophrenia and depression, in a Scottish family [48, 49]. DISC1 is a synaptic protein expressed in early development and plays a key role in neurogenesis, neuronal migration, and synaptic plasticity. However, genome-wide association studies have failed to identify DISC1 as a gene associated with schizophrenia, at least in the current diagnostic framework. Nonetheless, there are several mouse models of disrupted DISC1 function, by expression of mutant forms of DISC1, that display alteration in brain connectivity and function during developmental trajectory, which may underlie behavioral and cognitive phenotypes relevant to schizophrenia and ASD. Transgenic animal models that interfere with DISC1 function have shown a behavioral phenotype that is considered relevant for the human condition, accompanied by impairments at the anatomical, cell biological, and circuitry levels [49]. These models show dysregulation of different cell populations, including the best studied neuronal populations of the prefrontal cortex, but also astrocytes, oligodendrocytes, and CA1 pyramidal neurons. In this context, models may be useful to study cell interactions, as for example neuron–glia interaction. On the other hand, dominant-negative (DN)-DISC1 transgenic approach has linked deficits in cognitive and motivational behavioral paradigms with increased levels of oxidative stress, particularly in the prefrontal cortex [50]. This indicates that oxidative stress could be viewed as a cellular readout of the global level of stress in the individual [49, 50]. DISC1 inhibits glycogen synthase kinase (GSK)-3β signaling, a key signaling molecule in the immune response, that regulates important transcription factors, including nuclear factor κB (NFκB), which is linked to cytokine production [51]. In fact, DISC1 mutant animals had impaired GSK-3β signaling and higher levels of IL-1β and IL-5 in fetal brain [52].

### Neurodevelopmental models of ASD and schizophrenia

Epidemiological studies have suggested a link between prenatal or early postnatal stress or infection, and the development of neuropsychiatric disorders, in particular ASD and schizophrenia [53–55]. This led to the development of models for early life events that involve injury or stress, such as the neonatal ventral hippocampal lesion model and the social isolation rearing model. Lipska and Weinberger [56, 57] developed a neonatal lesion model that is triggered by microinjection of ibotenic acid, a toxin with excitatory effects, in the ventral hippocampus at postnatal day 7. This induces abnormal behavioral phenotypes akin to schizophrenia that become evident after puberty. These behavioral phenotypes include enhanced drug-induced locomotor hyperactivity, which can be reversed by anti-psychotics (e.g., haloperidol, clozapine) [56–58], conferring predictive validity to this model. These animals display persistent astrogliosis and microglial activation, including production of inflammatory mediators, accompanied by a significant increase in metabotropic glutamate receptor type 5 (mGluR5) expression within two distinct neuroinflammatory cell types: astrocytes and microglia [59]. The authors combined in vivo positron emission tomography (PET) analysis with post-mortem histological investigation, to determine the role of inflammation in neurotoxin-induced lesions. They show that hippocampal neuronal loss and glial mGluR5 expression, as well as some of the behavioral perturbations associated to the excitotoxic lesions, could be prevented by anti-inflammatory treatment with minocycline [59].

Prenatal stress or exposure to drugs is a known risk factor for neurodevelopmental disorders, such as schizophrenia and ASD. Prenatal stress exposure models and sex differences observed have been recently reviewed [58]. Among prenatal stress models, inescapable foot shock or corticosterone administration in the last trimester of pregnancy caused male-specific disruptions to latent inhibition at adulthood, while restraint stress had no effect in both sexes. Also, excessive glucocorticoid receptor stimulation, via administration of dexamethasone, caused an increase in pre-pulse inhibition (PPI) in both adolescent and adult offspring. Prenatal stress in rats caused a significant rise in serum corticosterone and forebrain NMDA receptor 2B. In terms of cognitive ability, male adult rats tend to be more affected by prenatal stress [58]. Moreover, pre-natal exposure to the anti-epileptic drug valproic acid (VPA) led to reduced social interaction in animals [60], and also in children [61, 62]. Rodents exposed to VPA have been used as a model for ASD [62]. Studies in these animals showed that male offspring had reduced social interaction, accompanied by increased neuroinflammation, enhanced glial activation, and altered systemic response to inflammatory stimulation [60], as well as some changes to serotonin levels and increased intestinal inflammation [63]. The same team has shown that although adult female animals were reported to have normal sociability, female pups displayed behavioral and inflammatory impairments [64]. Recently, functional genomic analysis in this model identified the inflammatory pathway, together with cognitive function and synaptic molecules, as differently expressed in VPA animals [65].

Following epidemiological studies showing that maternal infection by different pathogens, like bacteria, virus, or parasites led to increased incidence of schizophrenia and ASD, animal models of maternal immune activation (MIA) were developed. It has been documented that different infections (viral, bacterial, etc.) or just immune stimulation (lipopolisaccharide (LPS) or polyriboinosinic-polyribocytidilic acid, poly (I:C)) in the absence of infection, can lead to increased cerebral palsy (CP), ASD, and schizophrenia-relevant behaviors in the offspring, reviewed in [66]. In this context, it has been proposed that maternal cytokine-associated inflammatory response may be the link in the relationship between infections during pregnancy and the development of neuropsychiatric conditions [66]. The best characterized and most used models imply maternal gestational exposure to human influenza virus, the viral mimic poly (I:C), the bacterial endotoxin lipopolysaccharide, the local inflammatory agent turpentine, or exposure to selected inflammatory cytokines, like IL-6 [67]. MIA models have been reported as displaying the core symptoms of autism, reduced ultrasonic vocalizations (USVs) from pups to adulthood, decreased sociability, and increased repetitive or stereotyped behavior [68]. A previous study reported the influence of the maternal response on the offspring, indicating that pregnant dams that lost weight following MIA showed increased levels of TNF-α compared to controls [69]. Also, their offspring showed the most severe behavioral outcome, while offspring from dams that gained weight after MIA had no clear behavioral deficits [69]. Moreover, MIA induced by poly (I:C) appears to be dependent on the time of maternal immune activation, which can affect the pattern of symptoms that emerge later in life [70]. The interneuron populations involved in abnormal functional neurotransmission and their correlation with behavioral symptoms are now also being uncovered. A recent study used a combination of MIA and optogenetic inhibition to implicate parvalbumin (PV) expressing interneurons in medial prefrontal cortex, with the affective and cognitive symptoms observed [71].

MIA animal models have shown that cytokine imbalance in the fetal brain can result in neuroanatomical defects and behavioral abnormalities. This takes place independently of how cytokines that reach the fetal brain are produced: by the mother (crossing the placenta), by the placenta, or by the fetal brain itself [66]. Moreover, important roles have been reported for IL-6 in the immunological homeostasis of the fetal brain, and in the development of MIA-induced symptom in the offspring [72, 73]. A recent study by Choi and collegues elegantly shows the contribution of IL-6 and IL-17 [74]. The work combined the MIA model with KO mice, and the use of blocking antibodies. It pinpointed the retinoic acid receptor-related orphan nuclear receptor **γ**t (ROR**γ**t)-dependent effector T lymphocytes [e.g., T helper 17 (TH17) cells] and their main effector cytokine interleukin-17a (IL-17a) as necessary for abnormalities in the offspring of the MIA model [74]. The authors show that IL-6 upregulation in the dam’s serum is necessary and sufficient for the effects observed and the increase in IL-17a. Neither IL-6 nor other inflammatory cytokines were produced at the placenta/decidua level. Instead, levels of IL-17a were the ones that increased. IL-17a is also indicated as a potential therapeutic target in susceptible females [74].

The mouse maternal response to poly (I:C) leads to increased levels of serum pro-inflammatory cytokines like IL-1β, IL-6, and TNF-α, as well as the anti-inflammatory cytokine IL-10, which has parallel with increased serum levels of pro-inflammatory cytokines in mothers of children with ASD [66]. Among these cytokines, maternal IL-6 levels have been suggested to mediate the effects of MIA. A previous report showed that IL-6 injection in the dam was able to produce behavioral deficits in the adult mouse offspring, while IL-6 KO mice failed to produce several of the characteristic abnormalities upon MIA, suggesting that IL-6 elevation in the dam’s serum would suffice in mediating the effects on the offspring [75]. Rodent-based studies have been supporting a role for pre-natal poly (I:C)-induced MIA in the development of a panoply of behavioral, cognitive, and pharmacological dysfunctions [67]. In terms of face validity, numerous neurochemical and brain morphological abnormalities have been detected in adult mice and rats after maternal gestational exposure to poly (I:C). This model has been reported to have face and construct validity for ASD and schizophrenia, and predictive validity for schizophrenia. The offspring display behaviors that are consistent with schizophrenia and also with ASD, including elevated anxiety and deficits in PPI, latent inhibition (LI), and working memory. Some of these behaviors could be ameliorated by treatment with anti-psychotic drugs. Adult MIA offspring also exhibit abnormalities in gene expression and neurochemistry similar to those noted in schizophrenia and ASD. Finally, neuropathology is also seen in this model, including enlarged ventricles and a spatially localized deficit in Purkinje cells, characteristic of schizophrenia and ASD, respectively. The levels of cytokines in different areas of the brain and in serum of mice have been measured, comparing maternal intraperitoneal administration of poly (I:C) with saline injection [76]. The authors found that different pro-inflammatory cytokines were increased in areas of the brain in poly (I:C) offspring, when compared to controls. At day 0 post-birth IL-12(p70) was increased in frontal and cingulated cortex, but the frontal cortex also had large increases in IL-1β IL-6 and GM-CSF, while the cingulated cortex showed increased IFN-γ and chemokine (C-C motif) ligand 2 (CCL2, also known as MCP-1). O the other hand, in the hippocampus IL-1β showed the largest increase. The pattern of cytokine increase was not the same in different regions of the brain along time, and did not correlate with differences in serum. At the same time point (day 0) in serum granulocyte macrophage-colony stimulating factor (GM-CSF), IL-12 (p40), and CCL5 (also known as RANTES), among others were increased in the MIA offspring [76]. A persistent pro-inflammatory brain environment upon maternal exposure to poly (I:C) is evidenced in this study. Related findings have also been reported in non-human primate models of maternal infection and poly (I:C) MIA [77]. In vivo multiphoton microscopy shows that MIA offspring have impaired synaptic development, which persist until adulthood. Probably, these alterations could be prevented by the administration of an anti-inflammatory agent capable of crossing the blood-brain barrier, starting on day 1 after birth [78]. A recent report in the MIA model of schizophrenia shows TLR3 activation, oxidative/nitrosative stress, and increased pro-inflammatory mediators [79]. Chronic administration of paliperidone in young adults reverted TLR3 signaling activation, blocking neuroinflammation, stimulating M2 microglia polarization, and reverting cognitive deficits [79].

There is some controversy about the effects of MIA on fetal brain microglia. Some authors defend that MIA results in increased levels of microglial activation [80–82], and cytokine and chemokine expression in the fetal brain [73], while others share the view that MIA does not influence fetal microglia activation [69, 83, 84]. Flow cytometry has been used to analyze microglia activation in these animals, upon brain cell dissociation, investigating the developmental profile of these cells [85]. Also, a very recent study used a flow cytometry protocol for microglia to investigate levels of expression of CD11b, CD45, and Iba1 [86]. Different levels of expression of these proteins in mice that were born from poly:IC treated dams, when compared to saline treated females [86], were found. In another study using this model, microglia cells were activated in MIA offspring, resulting in increased expression of IL-1β and TNF-α [82]. Moreover, treatment with the antibiotic minocycline reduced cytokine production and rescued neurogenesis and behavior, supporting anti-inflammatory strategies in the treatment of schizophrenia [82]. The activation of microglia and its contribution to cytokine production and neuroinflammation are discussed in detail in the next section.

PET imaging was used to show that pharmacological activation of mGluR5 during 5 weeks reduced expression of classic inflammation marker PBR in many brain areas, and that this molecular association was not present in the offspring of LPS-exposed dams [87]. The post-mortem analysis revealed that the downregulation of PBR was mediated through activation of mGluR5 in astrocytes [87]. Activation of mGluR5 receptor appears as an important pathway in pre-natal and neonatal stress models [59, 87].

In a model of post-natal inflammatory stress, atypical anti-psychotics were evaluated for their ability to suppress the production of pro-inflammatory mediators in vivo, and in response to LPS challenge. The authors found that clozapine, olanzapine, and risperidone, but not haloperidol, suppressed TNF-α and IL-6, and upregulated IL-10, with clozapine promoting the highest increase in serum levels of IL-10. Clozapine also worked as anti-inflammatory in response to poly[I:C]-induced inflammation [88].

### Combining different models to better mimic ASD and schizophrenia

Combining different models and stimuli can give a more accurate construct validity to ASD and schizophrenia animal models. The influence of genetic factors (G), such as a gene mutation and environmental factors (E), like viral infection before birth, can be combined by, for example, performing pre-natal treatment with poly (I:C) in the DISC1 mutant animals. When the MIA model was performed in the inducible DISC1 knockdown mice, it led to more exacerbated schizophrenia phenotypes, compared with either single operation [67], and also a more exacerbated increase in IL-6 in fetal brain [89]. Another study performed the combination model in the inducible double negative hmDISC1 mutant, leading to no significant increase in IL-6 in the fetal brain, but high IL-1β, while GSK-3β signaling and IL-5, that should rise in response to polyI:C, did not increase [52]. This was accompanied by aggravation of some behavioral parameters, like sociability, while others, such as memory and PPI, were not affected [52]. The combined influence of genetic factors and MIA was investigated in another model, the neuregulin 1(NRG1) mutant mouse [90]. The authors cross-fostered the offspring between vehicle-treated or poly I:C-treated dams and evaluated schizophrenia-related behavioral characteristics at adolescence and in adulthood. Combining NRG1 disruption and MIA caused developmental stage-specific deficits in social behavior, spatial working memory, and PPI. Multiple combinations of mutant, MIA, and cross-fostering indicated that combining NRG1 deletion with both MIA exposure and cross-fostering had a robust effect on PPI [90].

The combination of two environmental factors (E x E) has also been used, particularly combining the MIA model with other post-natal stressors, like being reared by stressed mother [91], peripubertal stress [92], or adult immune challenge [60]. The contributions of pre-natal effects of MIA and the post-natal effects of being reared by an immune-challenged mother, and their contribution to the response to acute amphetamine challenge, have been investigated [91]. The results showed that parameters related to spatial memory were mediated by pre-natal, but not postnatal, maternal effects on the offspring. Nonetheless, being reared by an immune-challenged mother appears to constitute a risk factor for some behavioral and molecular abnormalities. Also, the results indicate that although the full-spectrum of cognitive abnormalities is only significant in the adult, some forms of cognitive impairment are already detectable in pubescent mice [91]. The combination of MIA with peri-pubertal stress led to neuropathological effects that were significant but restricted to the hippocampus GABAergic cell population [92]. A very elegant study found that MIA combined with peripubertal stress are synergistic in their pathological effects on adult behavior and response to immune challenges [93]. Evidence from animal models supports the two (or multiple) hit models for mental illness, particularly schizophrenia.

Most animal models discussed above highlight the importance of activated microglia and neuroinflammation during disease development. In the coming section, the contribution of microglial activation for the pathophysiology of schizophrenia and ASD will be discussed.

## Microglia: a key player in human neuroinflammation

The majority (80–90%) of cells in the brain are microglia and astrocytes [94]. Microglia are the primary immune cells in the central nervous system (CNS) and belong to the monocyte/macrophage lineage, having a myeloid origin [95]. Although repopulation of the brain by microglia originating in the bone marrow is debatable, it appears that migration of myeloid progenitors into the CNS is in the origin of resident microglia [96].

The role of microglia in CNS development includes a variety of complex functions, namely, (1) phagocytosis during neuronal/synaptic development, (e.g., by pruning of redundant neurons and connections) and removal of damaged cells, (2) recognition of pathogens, (3) antigen presentation, e.g., pathogen recognition (4) recognition of antibodies bound to pathogens, (5) cytotoxicity, through the secretion of reactive oxygen species and cytokines, (6) matrix remodeling, through production of matrix metalloproteinases (MMPs), (7) modulation of inflammation and immune responses, via the release of chemokines and IFN-γ, (8) repair, via the removal of cell debris, (9) regulation of stem cell proliferation, (10) response to neoplastic cells, (11) transport of lipoprotein, (12) viral entry into the CNS, e.g., entry of HIV into macrophages, (13) intracytoplasmic survival of mycobacteria and (14) demyelination [97]. The phenotypes of microglia are similar to those of peripheral monocytes/macrophages; while the M1 phenotype is responsible for the classical pro-inflammatory response, the M2 phenotype is associated with an anti-inflammatory, protective, behavior.

Astrocytes are the largest glial cell population in the brain. Both microglia and astrocytes are capable of producing pro- and anti-inflammatory cytokines and, therefore, are considered immunocompetent cells. Microglia, astrocytes, and the molecules they secrete have been recognized as central in the development of the nervous system. Their morphology and function accompany changes that occur throughout life, such as memory and learning, and have been associated with psychiatric disorders, such as depression and anxiety [94].

The following sections focus on abnormal aspects of microglia activity that are present in ASD and schizophrenia, highlighting aspects that are common to both diseases. The information available is summarized in Table 2.

**Table 2:** Microglia activation in ASD and schizophrenia

Feature	ASD	Schizophrenia	Proposed implication
General effects			
Overall deregulation of the immune system with increased production of pro-inflammatory cytokines, possible as a result of the activation of microglia	(Patterson, 2009; Rodriguez and Kern, 2011; Takano, 2015)	(Patterson, 2009; Takahashi et al. 2016)	Upregulation of iNOS, glutaminase, and inducible cyclooxygenase (COX-2) leading to an increase of nitric oxide (NO), glutamate and prostaglandins, respectively. These substances have a toxic effect in neurons(Fernandes et al. 2014)
Specific effects—data from analysis of biological materials			
Decrease in NK cell function, possibly as a result of increased production of NO by microglia	Enstrom et al., 2009)(Warren et al. 1987)	(Karpinski et al. 2016)	NK cells play important functions in innate immunity, sppecially against intracellular infections. Disfunction of NK cells may predispose to adverse neuroimmune interactions, namely, during development.
Glutathione (GSH) depletion, possibly caused by i-NOS increase	(Rodriguez and Kern, 2011)	(Ivanova et al. 2015; Zhang et al. 2016)	GSH (an antioxidant) has a protective effect on neurons. Its decrease may lead to easier neuron damage.
Increase in anti-phospholipid antibodies (APLAs)	Careaga et al., 2013)	NF	Increased risk of blood clotting and pregnancy losses
Denser distribution of microglia	In fronto-insular and visual cortex (Tetreault et al. 2012)	In pre-frontal white matter (Hercher et al. 2014). In frontal and temporal cortex(Garey, 2010)	Reduced number of neurons and/or disrupted neural connectivity
Increased levels of glial fibrillary acidic protein (GFAP) in the brain	Cerebrospinal fluid (Ahlsen et al. 1993). Cerebelum(Bailey et al. 1998). Anterior cingulate cortex white matter(Crawford et al. 2015)	Frontal cortex (Rao et al. 2013)	GFAP is an important protein in the central nervous system, in particular in repair after CNS injury. An increase in GFAP is a hallmark of reactive gliosis (Kamphuis et al., 2015), which follows trauma or injury.
Increases expression of NF-kB in activated microglia	(Young et al. 2011)	(Rao et al. 2013)	NF-kB plays a key role in inflammation, through its ability to induce transcription of pro-inflammatory genes.
Presence of HLA-DR positive cells	In serum(Lee et al. 2006; Mead and Ashwood, 2015)	Brain, post-mortem (Bayer et al. 1999; Garey, 2010; Radewicz et al. 2000; Rao et al. 2013)	HLA-DR is an immunohistochemical marker that is expressed in antigen presenting cells (B lymphocytes, dendritic cells, macrophages). It reacts with activated microglia cells.
Increase in calprotectin	Increased levels in feces(de Magistris et al. 2010)	Localization in microglia(Foster et al. 2006)	Calprotectin is a pro-inflammatory marker. Increased fecal levels are due to increased intestinal permeability.
In vivo data			
Activation of microglia found by PET	(Suzuki et al. 2013)	(Bloomfield et al. 2016)	Confirms the importance of neuroinflammation in ASD and schizophrenia revealed by studies based on the analysis of tissues

*NF* no studies found

### Activation of microglia in ASD and schizophrenia

In response to injury, microglia become activated and upregulated certain antigen receptors (e.g., CD11b) and those for cytokines (e.g., IL-1 and IFN-γ) and chemokines (CCL4 and CXCL1). Upregulation of cytokines, activation of microglia and astrocytes, and an overall deregulation of the immune system have been associated with both schizophrenia and ASD [98], although the distinctive features between both pathologies have not been clearly identified. Most studies focus on one of the diseases or on common features, in particular, those related to neuroinflammation of the brain and markers of this process. For instance, it is now clear that neuroinflammation of the brain and, in particular, activation of microglia, are associated with ASD [34, 99]. In vivo studies with positron emission tomography (PET) have confirmed the activation of microglia both in ASD [100] and schizophrenia [101]. Although inflammatory markers are present in brain samples of both diseases, studies that focus on microglia and brain cytokines show that only some markers are common to both diseases. In particular, IL-6, IL-8, and TNF-α are deregulated in brain samples of both diseases compared with controls [102, 103]. On the contrary, increased IL-1β levels in the brain have been associated with schizophrenia [103] but its levels are not significantly deregulated in autism brain samples [104].

Activation of microglia is translated into an increased production/expression of cytokines and chemokines and activation of inducible nitric oxide (NO)-synthase (i-NOS) [34, 99]. Upregulation of iNOS, glutaminase, and inducible cyclooxygenase (COX-2) leads to an increase of nitric oxide (NO), glutamate, and prostaglandins, respectively. Most of these factors released by activated microglia have a toxic effect in neurons [105]. Several studies have pointed out that an increase in NO leads to a decrease in NK cell function, which seems to be altered in children with ASD [106, 107]. A reduction in the number of NK cells was observed in patients with schizophrenia, which was not paralleled in bipolar disorder [108].

The i-NOS activity increase may be the cause for the reduction in glutathione (GSH) levels [109], and since GSH has a protective antioxidant effect in neurons [110], the latter would be damaged more easily. GSH depletion has been considered an important characteristic in children with ASD [34]. This decrease in GSH is accompanied by an increase in the concentration of oxidized glutathione (GSSG) in the cerebellum and temporal cortex of brain samples from patients with ASD [111], i.e., the redox ratio of GSH to GSSG was decreased by 52.8 and 60.8%, respectively. A reduction in GSH levels has also been observed in schizophrenia [112, 113]. A post-mortem study reported that the brains of patients with ASD and schizophrenia share a deficit of vitamin B12 [112]. This vitamin has a key role in the function of brain and nervous system, and is only synthesized by certain bacteria. Meat, fish, dairy, and eggs are common sources of this vitamin for humans.

Activation of the immune system may also lead to production of anti-phospholipid antibodies (APLAs). Increased levels of APLAs (e.g., anti-cardiolipin, β2-glycoprotein 1, and anti-phosphoserine), which are normally associated with increased risk of blood clotting and pregnancy losses, have been identified in the plasma of young (age range 24–82 months) children with ASD and associated with their impaired behavior [114].

Reversion of symptoms achieved through medication should lead to re-establishment of homeostatic conditions, i.e., downregulation of cytokines and other biomarkers, namely, those produced by microglia. However, as discussed in Section 4.2.1, treatment of schizophrenic patients with risperidone does not lead to a consistent pattern of cytokine variation. This calls for further investigations because it is not thus far possible to assign predictive value, in terms of diagnostic and/or clinical follow up, to a single biomarker.

Further insights into the role of microglia, the search for novel biomarkers and the importance of external stimuli (including medication), may be provided by animal models, as discussed in Section 2. In spite of the shortcomings of animal models to investigate these complex diseases, we know from other areas of research how important these models have been for developing novel diagnostic and therapeutic tools. In this particular instance, the BTBR mouse model replicates features, e.g., behavioral and immunological, that are characteristic of ASD [37, 38]. Of importance is the production of higher levels of pro-inflammatory cytokines (e.g., IL-6, CCL2, and CCL3 (also known as MIP-1alpha)) and lower levels of the anti-inflammatory cytokine IL-10 by macrophages stimulated by LPS [38]. Microglial activation and deactivation in these models may prove critical in setting a consistent pattern of variation of a core set of clinically useful inflammatory markers.

### Microglia in post-mortem studies of ASD and schizophrenia

Post-mortem studies have confirmed the increase in microglia activation [115, 116] in ASD, as well as reduced number of neurons in the fusiform gyrus [117], which is one of the cortical regions supporting face processing. A similar reduction in the number of neurons in the amygdala has been reported [118]. This may be related to the denser distribution of microglia that has been reported for the fronto-insular and visual cortex of patients with ASD [119]. If this distribution reflects a greater tendency towards a pro-inflammatory profile then an upregulation of pro-inflammatory signals is to be expected, as has in fact been reported [120]. Moreover, around 20 years ago, increased levels of GFAP were reported for autistic children [121, 122]. This may be taken as an indication of microglial and astroglial activation, with a concomitant increase in several pro-inflammatory markers, including TNF-alpha, IL-6, CXCL8, among others. This exaggerated brain immune response may be associated to the aberrant expression of nuclear factor kappa-light-chain enhancer of activated B cells (NF-**k**B) in microglia [123].

The first evidence of activation of microglia in schizophrenia was provided in 1999 [124]. The authors conducted immunohistochemical analysis on post-mortem frontal cortex and hippocampus of 14 patients with schizophrenia using the human major histocompatibility complex (MHC) class II antigen human leukocyte antigen-antigen D related (HLA-DR), which is expressed on professional antigen-presenting cells like dendritic cells, B cells, and monocytes/macrophages, as a marker. In 3 of the 14 patients, they found HLA-DR positive cells but no activation of microglia in controls. Foster el al [125] quantified calprotectin, a pro-inflammatory marker and a calcium-binding protein, reporting its elevation in schizophrenic patients compared to controls. Its levels were higher in these patients than in those with bipolar and depressive disorders. Further, calprotectin was found to be localized to microglia, suggesting that this protein is associated with microglia activation. In ASD, no similar post-mortem studies have been carried out but increased levels of fecal calprotectin has been reported and associated with increased intestinal permeability [126]. However, more recently, no difference was found between children with autism and non-autistic individuals [127].

The above changes in microglia activity are paralleled by morphological modifications in neurons [128]. Neocortical pyramidal neurons showed partial loss of dendritic spines and glutamatergic neurons a decrease in number. The reduction was very significant in the frontal and temporal association cortex, with schizophrenic patients having only 30% of that found in controls. Importantly, this reduction was not associated with age or death. In the same study [128], the number of microglia in patients was higher than in controls: 28% in frontal area and 57% increase in the temporal area. The authors defended schizophrenia as a neurodevelopmental disorder, which indirectly manifested by early changes in behavior and intellectual performance, before being clinically identified as a disease. They postulated that early insult or congenital errors might have led to the recruitment of microglia to the site of the injury [128], with these cells remaining resident through to adulthood. This hypothesis assumes that permanent damage is caused early in life and that the brain does not have enough plasticity to counteract the damage, which would persist after a prolonged period of time.

The above cellular modifications in the brain are accompanied by anatomical alterations, some of which have been recognized for a long time. Perhaps the most consistent change is the enlargement of the cerebral ventricles. However, other anatomical features appear to be present in the schizophrenic brain, namely, a decrease in gray matter in frontal, temporal, and parietal cortices compared with controls [129]. The loss of dendritic spines would reduce the number of glutamate receptors, namely, of the N-methyl-D-aspartate (NMDA) subtype, thus reducing the number of sites for the binding of glutamate, which is an important neurotransmitter in the mammalian brain.

Although the origin of the damage to brain tissue associated with schizophrenia has been placed very early in life, including before birth, the precise events that may have triggered the persistent inflammatory state have not yet been clearly identified. Mild acute inflammatory states of the mother, including influenza, have been suggested as triggers for abnormal activation of microglia and overexpression of inflammatory cytokines. This has been modeled in animals, particularly the MIA model, where maternal immune activation leads to imbalanced cytokine profiles and characteristics akin to ASD and schizophrenia in the offspring, as reviewed in Section 2. It is somewhat surprising that devastating illnesses, like schizophrenia and ASD, might have originated from a simple flu. However, one should not look at the effect of the contamination by microorganisms in the brain as being a replication of what occurs in other organs. A very striking example of the uniqueness of response of individuals to infection is described by Fellerhoff et al. [130]. Their work was prompted by the occurrence of serious pneumonia in two children, who developed into mental illness. One was later diagnosed with ASD and the other with schizophrenia. Both children presented very high antibody titers against the pathogen *Chlamydophila*. The authors further investigated if the DNA of *Chlamydophila* (or other *Chlamydiaceae*) was present in samples of the frontal cortex of brains of 34 patients with schizophrenia. The DNA of these bacteria was found four times more often in these patients than in controls. Since the primary target of *Chlamydophila* are monocytes, the hypothesis put forward by the authors is, that in the brain, microglia would also be the primary target since they result from the differentiation of a subpopulation of monocytes. The authors also argue that contamination had probably occurred prior to the onset of the disease, based on the observation that 4% of the population is permanently infected with *Chlamydophila pneumoniae* (*C. pneumonia*) [130, 131] and the incidence of infection in schizophrenic patients is substantially higher (40.3%) than in controls (6.7%) [131].

The molecular profile of the brain of schizophrenic patients may not be significantly different from that of individuals from ASD, at least judging from the set of data that have been published so far. The difference may reside on how the brain reacts to stressful events and, in particular, on how the innate immune system responds in each case. In this context, the later onset of schizophrenia might be related to some immunomodulatory ability of the brain, via the action of microglia, to keep inflammation in a dormant state. In spite of being speculative, this assumption finds some justification in the small genetic differences observed in brains from patients with schizophrenia and individuals with bipolar disorders [132]. Only 28 genes were expressed differently, while 1268 genes were commonly altered in both diseases. All these genes were overexpressed in bipolar patients with respect to schizophrenic subjects. Of note is that in the group of 28 genes which are expressed differently are those associated with microglial function, namely, triggering receptor expressed on myeloid cells (TREM)2, (toll-like receptor (TLR)1, TYRO protein tyrosine kinase-binding protein (TYROBP), C1QA, CD68, serpin family A member (SERPINA)1, CD14, and AIF1. Among these, the presence of CD68, one of the markers of microglial activation [133] is of particular importance. CD68 is also an important marker for activation of phagocytic cells in peripheral blood. In spite of genetic factors being possibly shared by these diseases [132], the intermediation of microglia in resolving environmental challenges to the brain appears to be at the core of these diseases. For reasons still unknown, the delicate balance between normalcy and pathology would be tipped at some stage in development by events that amplify a certain group of symptoms. Clinically, some of these symptoms are common to two or more diseases and their relative importance may vary throughout life. This argues in favor of an explanation of disease etiology essentially based on an unbalanced response to environmental stimuli (e.g., psychological, chemical, and microbial).

### Microglia in ASD and schizophrenia; in vivo studies

Studying the activation of microglia in live subjects is of great value, particularly to monitor the evolution of neuroinflammation in the brain with age and the effect of medication. This information, combined with the indirect assessment of glial activation via peripheral blood biomarkers, could provide a clear picture of the processes that are occurring in vivo.

With the advent of non-invasive methods of studying brain function, such as functional magnetic resonance imaging (fMRI) and PET, valuable information can be extracted. For instance, PET can be used to investigate activation of microglia in diseased brain [134]. The specific application of this technique to identify activated microglia is based on the ability of some markers, e.g., isoquinoline PK11195 (used in the form of a radiotracer: [^11^C](R)-PK11195), to selectively bind to microglia that are activated, but not to resting cells. Subtle glial responses occur in areas of the brain that are microanatomically unchanged, providing evidence for some plasticity of the injured brain [134].

The use of the first generation radio-tracers, like [^11^C](R)-PK11195, and limitations of PET scanners used in early studies have been questioned in comparison with second generation imaging agents and high resolution machines [135]. The major problems associated with [^11^C](R)-PK11195 have related to its relatively low uptake by the brain, associated with binding to organs in the periphery, and non-specific binding, related to its lipophilic nature [136]. Kenk et al. [135] argue for the need to incorporate genotyping information in the analysis of the PET scans, since this parameter is known to influence the binding of the mitochondrial translocator protein 18 kDa (TSPO) to second generation radioligands. TSPO is expressed by activated microglia [137].

In a study by Suzuki et al., the application of PET using [^11^C](R)-PK11195) revealed substantial activation of microglia in patients with ASD (age comprised between 18.6 and 31.9) [100]. However, the regional distribution was not different between these subjects and controls. No other studies dealing with in vivo imaging studies (e.g., PET and fMRI) on the activity of microglia in individuals with autism could be found.

No difference between patients with schizophrenia and controls when the new radiomarker [18F]-FEPPA and a high resolution research tomography were employed, both in gray and white matter brain regions has been found [135]. However, in a very recent study using a novel radio-ligand, [C-11]PBR28 [101] the activity of microglia in patients with schizophrenia was found to be augmented in comparison with healthy controls. This agrees with other findings reported using PET, namely, in the hippocampus of schizophrenic patients [138] and in total gray matter of the brain already within the first 5 years of disease onset [139].

## Clinical research: inflammatory cells and biomarkers in ASD and schizophrenia

### Biomarkers and clinical features in ASD and schizophrenia

#### Biomarkers related to immune function and oxidative stress

There is increasing evidence of altered immune function in ASD and schizophrenia, whether it is related to differences in populations of immune cells, in serum levels of antibodies, or levels of cytokines and interleukines. Such changes have been documented in other review papers [10–14], but evidence of how these changes associate with clinical features, and in particular disease severity, might further elucidate the pathophysiological mechanisms involved. Table 3 summarizes the main evidence from studies relating biomarkers of immune function and biomarkers of oxidative stress to clinical features in both ASD and schizophrenia.

**Table 3:** Biomarkers and clinical features in ASD and schizophrenia

Biomarker	Associated clinical features		References
	ASD	Schizophrenia	
Immune function			
IL-6	↑ in patients compared to controls; predominantly in children with regressive autism, associated with more impaired communication and aberrant behaviors; positive correlation with severity of autism and CARS scores		Ashwood et al. 2011; Yang et al. 2015 Chang-JiangYang et al. 2015
		↑ in patients compared to controls; correlated to negative symptoms and duration of illness	Kim et al. 2000
		State marker	Miller et al. 2013
TNF-α	↑ in patients compared to controls; positive correlation with the severity of autism; positively correlated to CARS scores		Yang et al. 2015 Chang-JiangYang et al. 2015
		↓ in patients compared to controls; negative correlation to PANSS total score, general psychopathology, positive and cognitive subscales	Lv, Tan, 2015; Noto, Maes, 2015a
		Trait marker	Miller et al. 2011
TNF-R1 and TNF-R2	NF	TNF-R1 significantly correlated with positive symptoms (PANSS)	Hope et al. 2013
		↑soluble forms, and associated with treatment resistance	Noto, Maes, 2015a
IL-1β	↑ in patients compared to controls; predominantly in children with regressive autism, associated with more impaired communication and aberrant behaviors		Ashwood et al. 2011
		State marker	Miller et al. 2011
IL-1RA	NF	Significantly correlated with negative symptoms (PANSS)	Hope et al. 2013
IL-12	↑ in patients compared to controls; predominantly in children with regressive autism, associated with more impaired communication and aberrant behaviors		Ashwood et al. 2011
		Trait marker	Miller et al. 2011
IFN-ϒ	NF	Trait marker	Miller et al. 2011
TGF-β	↓TGF-β1 in autistic children compared to controls or children with other DD; significant correlation with reduced adaptive behaviors and worse behavioral symptoms		Ashwood et al. 2008
		State marker	Miller et al. 2013
Chemokines	↑ osteopontin in autistic children compared to controls; positive correlation with CARS scores and disease severity		Al-Ayadhi and Mostafa, 2011
	↑ IL-8 in patients compared to controls; predominantly in children with regressive autism, associated with more impaired communication and aberrant behaviors		Ashwood et al. 2011
		↑ CCL11 and MIP-1α; CCL11 positively associated with negative symptoms ↑ MCP-1 associated with treatment resistance ↓ IP-10	Noto, Maes, 2015a
IL-2	NF	↓ in patients than controls; Inversely associated with negative symptoms; in patients negative correlation between IL-2 and total score in negative subscale of PANSS	Noto, Maes, 2015a; Azevedo et al. 2014
		↑ in patients compared to controls; significant inverse relationship with positive subscale of PANSS	Zhang et al. 2002
IL-2R	NF	Trait marker; ↑ in patients compared to controls; associated with Positive and Negative Syndrome Scale total scores, negative symptom and general psychopathology subscale scores	Miller et al. 2013; Bresee and Rapaport, 2009
Lymphocyte populations		↑ total lymphocytes, ↑T lymphocytes CD3+; ↑T helper CD4+; ↑CD4/CD8; ↓proportion T lymphocytes CD3+ in drug naïve FEP	Miller et al. 2013
	NF	↑ proportion of CD4+ and CD56+ in acutely relapsed patients; CD4/CD8 ratio in a state marker; CD56 is a trait marker	Miller et al. 2013
Immunoglobulins and antibodies	↓ plasma IgG and IgM in autistic children compared to other DD and healthy controls; correlated with behavioral severity in autistic children	NF	Heuer et al. 2008
	↑ anti-ganglioside M1 in autistic children compared to healthy controls; correlated with disease severity and CARS scores		Mostafa and Al-Ayadhi, 2011
	↑ anti-neuronal antibodies in autistic children compared to healthy controls; correlated with disease severity		Mostafa and Al-Ayadhi, 2012
Oxidative stress			
↓ pyridoxal	NF	Acute stage schizophrenia The greater the decrease in pyridoxal levels (admission to discharge) the less improvement in symptoms	Katsuta et al. 2014
↑ pentosodine ↓ pyridoxal	NF	Clinical features of treatment resistant schizophrenia; possible biomarkers	A rai et al. 2010; Miyashita et al. 2014

*NF* no studies found; *state marker* increased in acute phase; normalizes with treatment; *trait marker* remains elevated in acute phase and following treatment

A meta-analysis in drug-naïve patients with schizophrenia [140], reported increased numbers of total lymphocytes, T lymphocytes (CD3 positive), T helper cells (CD4 positive), and a higher ratio between T helper and T cytotoxic cells (CD4/CD8), but a reduced proportion of T lymphocytes. In acutely relapsed patients, the authors also found a higher proportion of CD4 positive and CD56 positive cells (T helper and natural killer, NK, cells, respectively). After treatment, the CD4/CD8 ratio decreased, while the concentration of CD56 positive cells increased, the former being a state marker while the latter a trait marker [140]. In a previous study, Miller et al. [11] suggested that cytokine levels were also associated with clinical status; some cytokines like IL-1β, IL-6, and TGF-β seem to act as *state markers* for schizophrenia (they are increased during the acute phase of psychosis and normalize with anti-psychotic treatment) while others, such as IL-12, IFN-ϒ, TNF-α, and sIL-2R, appear to represent *trait markers* as their levels remain elevated in acute exacerbations and do not remit after anti-psychotic treatment.

Several studies have shown that changes in serum levels of antibodies correlate with disease severity in ASD. Children with autistic disorder had significantly reduced levels of plasma IgG and IgM compared to children with other developmental delays (DD) and typically developing controls [141]. This reduction correlated with behavioral severity, i.e., patients with the most reduced levels of IgG and IgM scored highest in behavioral tests. Significantly higher serum levels of anti-ganglioside M1 antibodies and anti-neuronal antibodies have also been correlated with disease severity and Child Autism Rating Scale (CARS) scores [142, 143]. Children with ASD very frequently show alteration of biochemical pathways, such as the methylation pathway, which performs complex functions, vital to overcoming neurological inflammation. Mutations in pathways, such as the methionine cycle, the folate cycle, the BH4 (biopterin) cycle, and the urea cycle (which are all interrelated) can produce biochemical imbalances which are, at least in part, responsible for the immune imbalance found in children with ASD [144].

A number of studies have found that certain cytokines and interleukines correlate with clinical features (type and severity of symptoms, clinical status, course of illness) both in ASD and schizophrenia (Table 3). The comparison of plasma samples of children with ASD with age-matched typically developing children and children with other DD [145] showed significant increases in the plasma levels of a number of cytokines. In the ASD group, increased cytokine levels were predominantly found in children with a regressive form of ASD and were associated with more impaired communication and aberrant behaviors.

Other studies have linked interleukines to symptom dimensions (positive and negative) in schizophrenia. Specifically, IL-2 has been associated with both the negative [146] and positive subscale [147] of the Positive and Negative Syndrome Scale (PANSS). The soluble IL-2 receptor (sIL-2R) has also been associated with the PANSS total scores, negative symptom and general psychopathology subscale scores [148]. On the other hand, IL-6 [149] has been correlated to negative symptoms and duration of illness, while IL1-RA [150] has been significantly correlated with negative symptoms (PANSS). IL-6 and serotonin levels were increased in autistic patients compared to controls and had a positive correlation with autism severity [151]. In another study [152], the same authors found increased levels of IL-6 and TNF-α, and decreased diurnal variation of cortisol (VAR) in patients with autism compared to controls. Further, IL-6 and TNF-α levels positively correlated to CARS scores while VAR negatively correlated to CARS scores.

Other important components of immune function have also been studied in ASD and schizophrenia. Namely, there is evidence that TNF-α, TGF-β, chemokines, and osteopontin (OPN) are also associated with clinical features and disease severity, as indicated below.

TNF-α is associated with several inflammatory conditions. In patients with chronic schizophrenia, its levels were significantly lower [19] and showed correlations with PANSS total score, PANSS positive and general psychopathology subscores, as well as the cognitive subscale. Specifically, patients with lowered levels of TNF-α were more likely to have severe psychopathological symptoms even though no differences were found between schizophrenia subtypes or type of anti-psychotic treatment. TNF-R1 was also significantly correlated with positive symptoms of PANNS (Hope et al. 2013). A deficient production of TNF-α might be associated with dysfunction of thought, perception, and behavior in these patients.

TGF-β is one of the most important regulators of the immune response. [153] found significantly lower plasma TGF-β1 levels in children with ASD compared with typically developing controls and with children with other developmental disabilities. Importantly, such levels were associated with reduced adaptive behaviors and worse behavioral symptoms.

Chemokines are small cytokines with crucial roles in homeostasis, as they control the movement of leukocytes into the central nervous system, regulate neuronal cell migration, proliferation and differentiation, and are important in neuronal-microglia communication. Chemokines are frequently deregulated in disease and have been implicated in ASD and schizophrenia [154]. In a study, the diagnosis of schizophrenia was accompanied by a specific cytokine-chemokine profile [154], i.e., increased levels of CCL11, CCL3, soluble TNF receptors 1 and 2 (sTNF-R1 and sTNF-R2), and decreased levels CXCL10 (also known as IFN-ϒ-inducible protein 10 or IP-10), TNF-α, IL-2, and IL-4. Using five of these biomarkers (sTNF-R1 and sTNF-R2, CCL11, CXCL10, IL-4), the authors found a sensitivity of 70% and specificity of 89.4% for the diagnosis of schizophrenia. IL-2 was inversely associated with negative symptoms, while CCL11 was positively associated with negative symptoms. Further, treatment resistance was associated with increased levels of both TNF receptors (sTNF-R1 and sTNF-R2) and CCL2.

OPN is a phosphoprotein with important roles in inflammation, cell adhesion, chemotaxis, immune response, and protection from apoptosis, depending on its location and context. Levels of OPN in autistic children were significantly higher than those of matched healthy children [155] and correlated with disease severity and CARS scores.

Oxidative stress appears to be important in many neuropsychiatric disorders, including schizophrenia, and several biomarkers can be used to assess oxidative stress and anti-oxidative status. Oxidative stress, which leads to damage of proteins, lipids, and DNA, converts glucose and lipids to reactive carbonyl compounds, which in turn are converted to advanced glycation end products. The accumulation of such products is referred to as carbonyl stress.

Katsuta et al. [156] evaluated the role of carbonyl stress markers in acute stage schizophrenia and found that pyridoxal levels (or vitamin B6 which detoxifies reactive carbonyl compounds) were significantly lower in patients with schizophrenia compared to that in controls. Further, the greater the decrease in pyridoxal levels over time (from admission to discharge) the lesser the improvement in symptoms. In chronic schizophrenia, lower pyridoxal levels have shown correlation with more severe symptoms, especially positive symptoms [157]. Reduced pyridoxal levels and an increased in pentosidine have also been correlated with treatment resistance [158]. Glutathione deficit has also been suggested as an indirect biomarker of oxidative stress in early-onset schizophrenia [159]. Another study examined the peripheral blood mononuclear cells (PBMC) gene expression levels of pro-inflammatory transcription regulator NF-kB and the downstream enzymes iNOS and COX-2, important in inflammatory and oxido/nitrosative status, respectively, and found significant increases in first episode psychosis, in relation to healthy controls. Concomitantly, the NFkB inhibitory subunit IkBa was decreased in patients. Together, these results indicate a pro-inflammatory state of PBMC in first episode phychosis [160]. A very recent review and meta-analysis in first episode and early-onset schizophrenia, concluded that although the heterogeneity between studies is too great to allow an effective comparison, oxidative stress and inflammation are likely increased and lead to worse outcomes [161].

#### MicroRNAs: novel and promising biomarkers in schizophrenia and ASD

Human genome sequencing projects revealed that the large majority of our genome do not encode for proteins [162]. Surprisingly, throughout evolution the number of protein coding genes remained relatively stable in the different species, while the number of non-protein coding genes greatly increased [162]. The importance of these non-protein coding transcripts has recently been explored. Noteworthy, non-coding RNA are implicated in human evolution and cognition [162] and have important biological functions [163]. These observations led to a shift in the potential of human non-coding genes. This class of genes can be divided in long non-coding and small non-coding RNAs, depending on the size of gene transcripts [164]. Among the latter, microRNAs (miRNA), with approximately 22-nucleotides long, are the most commonly studied. MiRNAs are key regulators of gene expression at a post-transcriptional level, by binding to 3′untranslated regions (UTR), coding sequences or 5′UTR of target messenger RNAs (mRNAs), which leads to inhibition of translation or mRNA degradation [163]. Interestingly, a single miRNA is able to regulate the expression of several genes (coding and non-protein coding genes). Therefore, a single miRNA may simultaneously control key mechanisms of schizophrenia or ASD and regulate inflammatory pathways. Moreover, miRNA levels can be detected in body fluids, including blood, saliva, and urine [165]. Consequently, miRNAs have emerged as new, unconventional, and promising biomarkers for diagnosis and prognosis of mental disorders [166]. In this review, we will focus particularly on miRNAs that have been consistently reported as blood-associated biomarkers for schizophrenia or ASD (Table 4), and we will address their link with inflammatory pathways.

**Table 4:** Blood-associated microRNAs as diagnostic markers in autism and schizophrenia

Disease	Samples		microRNAs		Reference
	Type	Number	Downregulated	Upregulated	
Autism	Serum	55 patients; 55 controls	miR-151a-3p, miR-181b-5p, miR-320a, miR-328, miR-433, miR-489, miR-572, miR-663a	miR-101–3p, miR-106b-5p, miR-130a-3p, miR-195-5p, miR-19b-3p	Mundalil Vasu et al. 2014
	Peripheral blood	20 patients; 20 controls	let-7a, let-7d, miR-103a, miR-1228	miR-34b	Huang et al. 2015
Schizophrenia	Plasma	564 patients; 400 controls	NF	miR-130b, miR-193a-3p	Wei et al. 2015
	PBMC	90 patients; 60 controls	miR-432	miR-34a, miR-449a, miR-564, miR-548d, miR-572, miR-652	Lai et al. 2011
	Plasma	50 patients; 50 controls	NF	miR-7	Zhang et al. 2015
	Plasma and PBMC	25 patients; 13 controls	NF	miR-132, miR-195, miR-30e, miR-7 in plasma; miR-212, miR-34a, miR-30e in PBMC	Sun, Lu et al. 2015a
	Plasma	61 patients; 62 controls	NF	miR-181b, miR-30e, miR-346, miR-34a, miR-7	Sun, Zhang et al. 2015b
	Serum	145 patients; 40 controls	miR-195, miR-17	miR-181b, miR-219-2-3p, miR-1308, let-7g, miR-346, miR-92a	Shi et al., 2012
	Plasma	20 patients; 20 controls	NF	miRNA-181b, miRNA-30e, miRNA-34a, miRNA-7	Song et al. 2014a

*NF* not found, *PBMC* peripheral blood mononuclear cells

The first hypothesis of miRNA involvement in schizophrenia was launched in 2005 [167], following publications in the cancer research field reporting miRNA direct control over tumor suppressors/oncogenes and the consequent impact on tumor development [163]. However, it was only in 2007 that the first scientific evidence associating miRNAs to schizophrenia emerged [168]. Perkins et al. reported that seven miRNAs were downregulated in postmortem prefrontal cortex tissue of individuals with schizophrenia compared with non-psychiatric disease individuals [168]. Three of those miRNAs, namely, miR-7, -212, and -132, were confirmed by an independent study [169]. Importantly, miR-7 overexpression has been identified in plasma samples from schizophrenic patients, compared with healthy controls [170]. This result has been validated by others, which strengthens miR-7 as a new diagnosis marker for schizophrenia [171, 172]. The role of miR-7 in regulating SHANK3 mRNA was uncovered using a hippocampal neuronal cell line, transduced with lentivirus for expression of miRNA mimics or its inhibitors [170, 173]. SHANK3 protein is expressed in cortex and hippocampus and contributes to synaptic development [173]. Similarly to humans, SHANK3 has been identified in genetic mice models of ASD [40], as highlighted in Section 2.1.1. Functionally, miR-7 overexpression decreased the density of dendritic spines in a SHANK3-dependent manner [40, 174]. This could partially explain miR-7 effect in neuropsychiatric disorders [170, 174]. However, considering schizophrenia as a multifactorial disease, it would not be surprising that miR-7 would also regulate inflammatory states. In dendritic cells cultures, miR-7 was found to be upregulated in activated dendritic cells stimulated with LPS and IFN-γ compared with both immature and tolerogenic dendritic cells, stimulated with GM-CSF and IL-4 or IL-10 and TGF-*β*, respectively [175]. miR-7 has also been found to be upregulated in chronic inflammatory and autoimmune diseases, including in nasal mucosa of patients with allergic rhinitis [176] and in B cells of systemic lupus erythematosus patients [177]. Wu et al. showed that impairment of B cell regulation is caused by a decrease in phosphatase and tensin homolog (PTEN) expression, which is post-transcriptionally regulated by miR-7 [177]. Notably, PTEN mutations have been widely described in ASD and loss of PTEN in Purkinje cell has been shown to induce autistic-like traits in mice [178]. Also, PTEN conditional KO mice revealed neurons with defective synaptic function, enlarged neuronal somata, and increased dendritic spine density, and also uncovered defects in myelination in the corpus callosum [179]. However, a direct contribution of miR-7/PTEN link to ASD remains to be determined. Deregulation of miR-7 expression levels in plasma or serum of patients with ASD has not yet been described.

Additionally, miR-212 and miR-132, which are transcribed from the same primary-miRNA and share the same seed region and some of the targets [180], have been widely reported as biomarkers for schizophrenia [168, 169]. miR-212 is upregulated in peripheral blood mononuclear cells from schizophrenic patients compared with healthy controls, while increased expression of miR-132 was found in plasma samples of schizophrenic patients [171]. Interestingly, miR-132 levels were decreased upon treatment [172]. Both miR-212 and miR-132 are able to simultaneously regulate neurons morphogenesis and immune processes [180]. On the one hand, mice with floxed-Cre and knockout alleles for miR-212/132 have dendrites with reduced length and branches in newborn neurons in the adult hippocampus [181]. Moreover, these miRNAs participate in the synaptic function [180]. miRNA-212/132 play a role in immune system regulation [180]. For instance, miRNA-212/132 levels are increased in inflammatory diseases such as rheumatoid arthritis and osteoarthritis [182, 183]. Supporting the role of these miRNAs in inflammation, miR-212/132 are induced by ligands of TLR1, TLR2, and TLR5 [182]. Therefore, miRNA-212 and miR-132 are potential schizophrenia markers and may mediate the interplay with inflammation. Regarding ASD, studies performed in lymphoblastoid cell lines derived from autistic patients and controls identified miR-132 as a biomarker [184, 185]. However, results are contradictory as one study reports miR-132 as overexpressed [184] while another shows miRNA-132 as underexpressed in patients versus controls [185].

miR-181b was shown to be upregulated in postmortem cortical gray matter from the superior temporal gyrus in schizophrenia [186]. This miRNA directly targets glutamate ionotropic receptor AMPA type subunit 2 (GRIA2), which has been implicated in development of synaptic plasticity, and the calcium sensor protein named visinin like 1 (VSNL1) [186]. Shi et al. reported miR-181 was upregulated in plasma samples of patients with schizophrenia compared with healthy controls [187]. This result was further confirmed by Song et al. [188]. Importantly, decreased expression of this miRNA was associated with symptomatology improvement after anti-psychotic treatment [188]. Furthermore, after patients’ stratification, it was found that serum levels of miR-181b were significantly increased in family schizophrenia than in sporadic schizophrenia [187]. In one of the first studies analyzing miRNAs as ASD biomarkers in plasma, samples of 55 autistic children and equivalent number of controls were tested [189]. In contrast to reports in schizophrenia, authors found miR-181b as significantly downregulated in autistic children compared with control group, and this result was independent of the method used for data normalization [189]. Therefore, miR-181b may have distinct roles in schizophrenia and ASD. However, the role of miR-181 in ASD is still inconsistent. Olde et al. used a pharmaceutical rat model of ASD, based on prenatally delivery of valproic acid, to study differently expressed miRNAs in amygdala, which is known to be enlarged in ASD patients [190]. Authors found significantly elevated levels of miR-181c, but not miR-181b, in the amygdala of valproic acid-exposed rats [190]. Experiments performed in microglia cell culture shown that miR-181c levels are decreased, while TNF-α levels are increased, when cells are cultured under oxygen-glucose deprivation [191]. Future studies on biomarkers should include all mature forms of miR-181 family. Hutchison et al. reported that miR-181 directly targets MECP2, a protein whose deregulation causes behavior disorders [192] (details on MECP2 in vivo animal studies are described in Section 2.1). Validation of miR-181:MECP2 targeting in independent studies is still missing. Mutations causing decreased levels of MECP2 have been associated with patients with Rett syndrome, which often exhibits ASD-like behaviors, while extra copies of this gene cause MECP2 duplication syndrome characterized by autistic behaviors [193]. Recently, Sztainberg et al. showed that symptoms of MECP2 duplication in mice were highly improved by restoration of normal MeCP2 levels using antisense oligonucleotides, which act through a base-pair complementarily mechanism similar to miRNAs [193]. Therefore, future studies using miR-181 for diagnosis or as a tool to understand molecular mechanisms in ASD and schizophrenia should be considered. In inflammation, the role of miR-181 also seems to be dependent on tissue/cell type. miR-181a, closely related to miR-181b, increases number of B cells by targeting a pro-apoptotic gene, BIM, in lymphoma cells [194], while in T cells it impairs cell sensitivity, and positive and negative selection [195, 196]. In fibroblasts, miR-181a directly regulates CXCL8, a pro-inflammatory cytokine [197]. Furthermore, in endothelial cells, miR-181b acts as an anti-inflammatory mediator by inhibiting NF-κB signaling pathway through targeting of importin-α3 [195, 198]. In vivo studies revealed that miR-181-deficient mice showed impaired lymphoid development and T cell homeostasis, and absence of mature NKT cells [199]. These effects in miR-181-KO mice were mediated by deregulation of PTEN levels [199], the gene commonly associated with comorbid ASD [200]. Finally, miR-181c has been implicated in neuroinflammation by targeting TNF-α in microglial cells [191]. The potential of miR-181 as a biomarker and as a molecular player in schizophrenia and ASD and the common traits to inflammation are worthy to be explored.

Another miRNA biomarker commonly described in schizophrenia is miR-30e, which is upregulated in plasma samples of schizophrenia patients compared to controls [171]. The same research group further validated miR-30e as a biomarker in a second cohort of patients and demonstrated its implication in the clinical outcome as miR-30e levels were decreased upon treatment [172]. Moreover, levels of this miRNA in peripheral blood mononuclear cells collected from schizophrenia patients were also increased, although miR-30e plasma levels have a higher sensitivity for diagnosis [171]. In ASD, miR-30a*, a miR-30 family member, was found to be differently expressed between cultured lymphoblastoid cells derived from autistic patients or their unaffected siblings, but so far, it has never been reported as an ASD biomarker in circulating plasma [201]. Importance of miR-30 for ASD is reinforced by the finding that miR-30 levels are increased in the amygdala following exposure to valproic acid, a rat model of ASD [190]. Furthermore, miR-30a is present in human prefrontal cortex and it targets BDNF, a crucial protein for cortical development and maturation [202]. Besides the role in neurons [202], miR-30 family is a player in the regulation of inflammation-associated genes [203, 204]. miR-30b attenuates phagocytosis and modulates secretion of TNF-α, IL-6, and IL-12p40 [203], while miR-30b contributes to B-cells dysfunction [205].

Independent studies reported increased levels of miR-34a in plasma samples of schizophrenia patients compared with healthy controls [172, 188]. High levels of this miRNA have also been found in peripheral blood mononuclear cells isolated from patients with schizophrenia [214]. Interestingly, levels of miR-34b, which mature sequence differs from miR-34a in only few nucleotides, are increased in peripheral blood of autistic patients [206]. However, the effect of miR-34a in inflammation is still controversial. On the one hand, miR-34a is associated with an anti-inflammatory profile by driving macrophage polarization into the M2 phenotype. This miRNA decreases levels of pro-inflammatory cytokines, including TNF-α and IL-6, and it inhibits pro-inflammatory response by decreasing notch1 levels and NF-kB activation in macrophages upon stimulation with lipopolysaccharide [207]. On the other hand, in a mouse macrophage cell line, miR-34a targets Twist-2 [208], which promotes production of the anti-inflammatory cytokine IL-10 in myeloid cells [209].

Other circulating miRNA biomarkers in schizophrenia and autism have been reported, but more consistent data is still needed to determine their significance as circulating biomarkers in the blood. For instance, the levels of miR-195 should be further investigated. This miRNA has been identified as upregulated in plasma of schizophrenia patients [171] and in serum of ASD patients [189], but other studies show it is decreased in serum of schizophrenia patients [187] or do not found a significant association [172]. Also, miR-195 levels are increased in plasma of schizophrenia patients resistant to treatment and decreased in patients responsive to treatment when compared with health donors [210] Within the brain, expression of this miRNAs is more associated with neurons rather than with glial cells [211]. Similarly to miR-30a, miR-195 directly targets BDNF [202].

Moreover, miR-346 was identified as a schizophrenia biomarker in plasma samples [172, 187], while its levels were only slightly increased in serum samples [187]. This miRNA regulates the release of the pro-inflammatory cytokine TNF-α, following stimulation of a monocytic cells line with LPS [212]. Additional potential miRNA biomarkers in schizophrenia include miR-219-2-3p, miR-1308, miR-92a, miR-17and let-7g in serum [187], miR-130b and miR-193a-3p in plasma [213], and miR-449a, miR-564, miR-432, miR-548d, miR-572, and miR-652 in monocytes [214]. Regarding ASD blood-associated biomarkers, further candidates include miR-151a-3p, 320a, miR-328, miR-433, miR-489, miR-572 as underexpressed, and miR-663, miR-101-3p, miR-106b-5p, miR-130a-3p, and miR-19b-3p as overexpressed in plasma samples [189]. Finally, a study in the Chinese population revealed that levels of let-7a, let-7d, miR-103a, and miR-1228 are reduced in peripheral blood of ASD patients compared with healthy controls [215]. Studies reporting circulating miRNAs as biomarkers for mental diseases are mainly restricted to plasma/serum or blood cell samples. Analysis of miRNA levels in urine as diagnostic or prognosis tool for schizophrenia or ASD has not been reported so far. However, a recent study was conducted in saliva of ASD patients, which showed differences in levels of a set of miRNA for ASD children compared with control group [216]. miRNA stability through association with proteins, and miRNA protection through encapsulation in extracellular vesicles (e.g., exosomes) are essential to avoid miRNA degradation and to allow the detection of miRNA in cell-free liquid biopsies [217].

Non-coding RNA research field is expected to continue to grow in the upcoming years and may shed light into the common traits between inflammation and behavioral disorders, including schizophrenia and ASD. Validation of reported miRNA biomarkers as well as search for novel and reliable miRNA candidates for schizophrenia and ASD diagnosis or prognosis are essential. Importantly, studies screening for inflammatory biomarkers should consider patients’ pharmacological treatments, as detailed in the next sections.

### Biomarkers and anti-psychotic treatment in ASD and schizophrenia

First-generation anti-psychotics (FGA) are known for their affinity to block dopamine D2 receptors and are effective in the treatment of psychotic symptoms (positive symptoms such as delusions and hallucinations) in illnesses such as schizophrenia. Their side effects are linked to their strong D2 antagonism throughout the brain and the blockade of muscarinic cholinergic, histamine, and alfa1 adrenergic receptors. Second-generation anti-psychotics (SGA or atypical anti-psychotics) differ from the FGA in their pharmacological properties and affinity for receptors. These drugs not only block serotonin and dopamine receptors and are equally effective in treating positive psychotic symptoms but also improve negative symptoms of schizophrenia. Also, the simultaneous action on both dopamine and serotonin receptors in different brain areas is responsible for their fewer side effects on movement and prolactin levels. However, SGA have a complex mixture of pharmacological properties and interact with multiple receptors for dopamine, serotonin, muscarinic cholinergic, histamine receptors, and alfa adrenergic receptors. They also have the capacity to block norepinephrine and serotonin reuptake in neurons. Overall, this complexity of pharmacological properties can lead to undesirable side effects which are clinically important and may contribute to patient non-compliance [218].

#### Effects of anti-psychotics on biomarkers of immune function and oxidative stress

The neurotransmitter dopamine not only regulates numerous bodily functions (behavior, movement, endocrine, cardiovascular, renal, and gastrointestinal function) but also constitutes an important bridge between the nervous and immune systems [219] since dopamine receptors are present in almost all immune cell subpopulations [220]. Dopamine (or dopamine agonists) have been shown to modulate the activation, proliferation, and cytokine production in immune cells [220, 221]. In a recent study by Yan et al. [219], the authors found that dopamine inhibits the NLRP3 inflammasome through the dopamine D1 receptor. The NLRP3 inflammasome is a cytosolic protein complex which is assembled in response to microbial infection or endogenous danger signals, thereby promoting the maturation and release of pro-inflammatory cytokines like IL-1β or IL-18. It is crucial in the initiation of inflammation and development of immune responses and has been implicated in diseases such as type 2 diabetes, atherosclerosis, and gout [222]. Yan et al. [219] not only demonstrated that dopamine is an endogenous inhibitor of NLRP3 activation but also suggested that dopamine is a potent anti-inflammatory agent.

On the other hand, serotonin (5-hydroxytryptamine (5-HT)) not only regulates many important physiologic aspects in the central nervous system (mood, aggression, sleep, appetite) but also peripherally (pain sensation, bone mass, tissue regeneration, platelet coagulation, and gastrointestinal function) [223]. Only 5% of the body’s 5-HT is produced in the brain. Platelets carry 5-HT, and th us are the major source of 5-HT for immune cells and lymph tissue, but monocytes, mast cells, and T cells also appear able to synthesize small amounts of 5-HT [223]. There is evidence that 5-HT has diverse signaling roles in immune cell function and is important in both innate and adaptive immune response. Further, 5-HT has been proposed as a T-cell modulator for many years now, and there is increasing evidence supporting the role for 5-HT signaling in B lymphocytes [224].

As described above, it is easily anticipated that antipsychotic drugs will most certainly affect immune function and the effects of anti-psychotics on cytokine levels have been documented in several studies Table 5 summarizes the main evidence from studies that relate biomarkers of immune function and oxidative stress to anti-psychotic treatment.

**Table 5:** Biomarkers and anti-psychotic treatment in ASD and schizophrenia

Biomarker	Associated clinical features		References
	ASD	Schizophrenia	
Immune function			
IL-6	NF	↑ in drug-naïve FEP patients compared to healthy controls; ↓ after 10-week treatment with risperidone; ↓ in patients with depressive symptoms (but not in those without); clozapine treatment appears to ↑IL-6; treatment with typical anti-psychotics ↓ IL-6 and sIL-6R; ↓after anti-psychotic treatment in relapsed patients ↑IL-6R in patients compared to controls	Noto et al. 2015; Song et al. 2014b; Tourjman et al. 2013;Maes et al., 1995; Maes et al. 1997; Borovcanin et al. 2013; Drzyzga et al. 2006
TNF-α	NF	↑ in drug-naïve FEP patients compared to healthy controls; ↓ after 10-week treatment with risperidone; ↓ in patients with depressive symptoms (but not in those without)	Noto et al. 2015
		Anti-psychotic treatment reported to have no effect or ↓ levels of TNF-α; clozapine reported to ↑ TNF-α	Tourjman et al. 2013; Meyer et al. 2011; Drzyzga et al. 2006
sTNF-R	NF	Clozapine treatment appears to increase sTNF-R	Tourjman et al. 2013
IL-1β	NF	↑ in drug-naïve FEP patients compared to healthy controls; after risperidone treatment levels returned to baseline at 6 months; ↓levels after anti-psychotic treatment	Song et al. 2014b; Tourjman et al. 2013; Meyer et al. 2011
IL-1RA	Levels did not change after 8-week treatment with risperidone despite clinical improvement	↑ drug-naïve FEP patients; ↓ after 6-week treatment with risperidone or olanzapine anti-psychotic treatment reported to have no effect on IL-1RA; clozapine treatment↑ IL-1RA; ↑ sIL-1RA with anti-psychotic treatment	De Witte et al. 2014; Tourjman et al. 2013; Maes et al. 1997; Meyer et al. 2011; Tobiasova et al. 2011
IL-12	NF	Possibly ↑ with anti-psychotic treatment	Tourjman et al. 2013
IFN-ϒ	Levels did not change after 8-week treatment with risperidone despite clinical improvement	↓ after anti-psychotic treatment	Tourjman et al. 2013; Tobiasova et al. 2011
TGF-β	NF	↑ in un-medicated FEP and schizophrenia relapse patients; further increased after treatment with anti-psychotics in FEP; unaffected by anti-psychotic treatment	Borovcanin et al. 2013; Tourjman et al. 2013
Chemokines	↓ Eotaxin and MCP-1 after 8-week treatment with risperidone; MCP-1 levels did not change after 8-week treatment with risperidone despite clinical improvement in another study		Choi et al. 2013; Tobiasova et al. 2011
	↑EGF in children with ASD; levels did not change after 8-week treatment with risperidone despite clinical improvement		Tobiasova et al. 2011
		↓CC16 in patients compared to controls; increase after treatment with clozapine	Maes et al. 1997
		↑S100B in drug-naïve and medicated patients compared to controls and also in drug-naïve compared to medicated patients; ↓S100B with haloperidol and clozapine	Zhang, Xiu, 2010a; Zhang et al. 2010a (VER)
IL-2	NF	Unaffected by anti-psychotic treatment; ↓ by first and second generation anti-psychotics	Tourjman et al. 2013; Drzyzga et al. 2006
IL-2R	NF	Increased in younger patients; treatment with clozapine increases sIL-2R levels	Maes et al. 1994
IL-10	↑ drug-naïve FEP patients; ↓after treatment with risperidone or olanzapine; changes in IL-10 correlated with improvements in negative, general and total symptom scores; another study reported no effect of anti-psychotics on IL-10		De Witte et al. 2014; Noto et al. 2015; Tourjman et al. 2013
IL-4		↓ after 10-week treatment with risperidone; another study reported no effect with anti-psychotics; ↓ after anti-psychotic treatment in FEP and relapse patents	Noto et al. 2015; Tourjman et al. 2013; Borovcanin et al. 2013
IL-15		↑ drug-naïve in FEP	De Witte et al. 2014
IL-13, IL-17, IL-1	↓ IL-13 in children with ASD compared to controls; levels of IL-13, IL-17, and IL-1 did not change after 8-week treatment with risperidone despite clinical improvement	IL-13 possibly ↓ by anti-psychotic treatment	Tobiasova et al. 2011; Tourjman et al. 2013
IL-27		↓ after anti-psychotic treatment in FEP	Borovcanin et al. 2013
Oxidative stress			
SOD and NO levels	NF	↑in patients with schizophrenia compared to controls Risperidone and haloperidol ↓ superoxide dismutase levels (but not nitric oxide levels) ↓ SOD levels at baseline predicted greater symptom improvement during treatment and greater change in SOD was correlated with greater symptom improvement	Zhang et al. 2012b
PON1 activity, TRAP, and LOOH levels	NF	↓PON1 activity and ↑TRAP in FEP ↑PON1 activity and↓ LOOH levels after 11 weeks of risperidone treatment	Noto et al. 2015b

*NF* no studies found

Results are somewhat heterogeneous across studies, and this may result from differences in stage of illness (first episode psychosis or chronic schizophrenia), schizophrenia subtypes, gender, smoking habits, age, medical comorbidities, or differences in anti-psychotic treatment (type of anti-psychotic, dosage and duration of treatment) [154].

Witte et al. [225] found a mixed pro- and anti-inflammatory profile in drug naïve FEP patients, that is, levels of interleukin IL-1RA, IL-10, and IL-15 were increased significantly compared to controls. The levels of IL-1RA and IL-10 decreased after treatment with atypical anti-psychotics, and the changes in IL-10 levels were significantly correlated with improvements in negative, general, and total symptom scores. The finding that only IL-10 responded to treatment in parallel with symptom improvement suggests that it could be used as a potential treatment response biomarker in future studies of schizophrenia.

In another study with drug naïve FEP patients [226] treatment with risperidone had a significant suppressant effect on several serum cytokine levels, namely, IL-6, IL-10, and TNF-α. Results suggest that risperidone seems to normalize a specific cytokine profile in first episode psychosis, characterized by monocytic and T-regulatory cell responses and additionally decrease Th2 functions. Curiously, the improvement in psychopathology with risperidone treatment was not related to risperidone-induced changes in cytokine levels from baseline to endpoint. Also, treatment with risperidone significantly decreased IL-4 and TNF-α but only in patients with depressive symptoms [226]. In contrast, Song et al. [227] found that in a sample of drug-naïve FEP, IL-6 returned to baseline levels but TNF-α increased following treatment with risperidone. Other studies have also found different effects on cytokine levels following risperidone treatment which might be explained by differences in media (immune cells versus microglia), study type (in vitro versus in vivo), stages of illness, and schizophrenia subtypes [154].

A recent meta-analysis on the effect of anti-psychotic drugs on blood levels of cytokines in patients with schizophrenia [228] found that haloperidol and clozapine treatment increases peripheral sIL-2R levels [11, 229], but leads to decreases in IL-1β and IFN-ϒ, and possibly increases in IL-12. Generally unaffected by anti-psychotic treatment are IL-2, IL-4, IL-6, IL-10, IL-1RA, sIL-6R, TGF-β1, and TNF-α, with the exception of clozapine which appears to increase IL-6 and sTNF-R. Typical anti-psychotics have been shown to suppress plasma IL-6, soluble IL-6 receptors, and transferrin receptor levels [230] whereas clozapine has been shown to increase sIL-2 receptor, IL-6, and IL-1RA [231, 232]. However, Borovcanin et al. [233] reported that treatment with anti-psychotics might decrease IL-4, IL-6, and IL-27 levels in schizophrenia.

Anti-psychotic drugs have also been noted for their potential anti-inflammatory role in schizophrenia. Consistent with the hypothesis that schizophrenia is associated with an exacerbation of the inflammatory response is the observation that the long-term administration of anti-psychotic drugs increases the level of anti-inflammatory cytokines, such as IL-10, and decreases the concentration of pro-inflammatory cytokines, namely, IL-1beta and TNF-alpha [234]. It is important to clarify if there is a difference between the effect of first and second generation anti-psychotic drugs [229]. Common to both groups appears to be the decrease of IL-2 levels (in vivo, ex vivo, and in vitro studies) [229] and a discernable difference between both groups on the serum levels of cytokines does not appear to exist. Clozapine, contrary to typical (haloperidol), and other atypical (olanzapine and risperidone) anti-psychotics stimulates the levels of IL-6 and TNF-alpha [229], which might be related to some of its adverse effects. In summary, anti-inflammatory and anti-psychotic drugs appear to act as modulators of the inflammatory response, presumably by decreasing the activity of microglia, which would manifest itself by an inhibition of pro-inflammatory cytokines and the prostaglandin PGE2 production.

Only two anti-psychotics (risperidone and aripiprazole) have been approved by the FDA for treatment of irritability in ASD, but neither drug shows improvement in the core symptoms of the disorder, and the long-term consequence of their use in children remains unknown. Therefore, few studies are available on the effects of anti-psychotics on biomarkers of immune function in ASD. Tobiasova et al. [235] evaluated whether risperidone-associated improvement was related to changes in concentrations of inflammatory cytokines and growth factors (EGF, IFN-ϒ, IL-13, IL-17, CCL2, IL-1, IL-1-RA) in children with ASD. This study found that although ASD patients had increased levels of EGF and decreased levels of IL-13 compared to controls, all inflammatory serum markers remained stable over a period of 8-week treatment with risperidone. Interestingly, even though treatment induced clinical improvement in aberrant and maladaptive behavior, this was not associated with changes in serum levels of these inflammatory biomarkers. However, in a study by Choi et al. [236], there was a significant decrease in CCL11 (also known as Eotaxin) and CCL2 levels after 8 weeks of risperidone treatment in children with ASD. Also, mean values of IL-5 were significantly higher in the responder group compared to non-responders.

The evidence of microglial activation in schizophrenia has been addressed in Section 3 of this review, and the indirect assessment of glial activation via peripheral blood markers is important for understanding the processes that are occurring in vivo. Glial cell activation stimulates astrocytes to produce SB100, which is a marker of inflammation. Increased S100B levels in the early stages of schizophrenia support the idea of neurodegenerative process and anti-psychotics such as haloperidol and clozapine have been shown to decrease S100B release from glial cells [237]. S100B levels were assessed in the serum of drug-naïve early-stage, medicated chronic schizophrenia patients, and healthy controls [237]. Results showed significantly increased serum S100B levels in both drug-naïve and medicated patients compared to controls, and also in drug naïve compared to medicated patients.

In vitro studies regarding the release of inflammatory markers, under the influence of anti-psychotic drugs, shed some light on the above results. For instance, LPS-activated glial cells showed a reduced level of IL-1beta and IL-2 after administration of chlorpromazine [238]. Risperidone appears to be particularly effective in inhibiting the activation of microglia by iNOS [239], which, as discussed earlier, is detrimental to neurogenesis. Haloperidol and risperidone inhibited the secretion of S100B by glioma cells, after stimulation by IL-6 [240]. This is in accordance with the finding that S100B levels were significantly higher in drug-naïve patients than in those that were medicated, as discussed earlier.

While there is evidence that oxidative stress pathways are involved in the pathophysiology of schizophrenia, there is less information regarding the effects of anti-psychotics on these pathways. Zhang et al. [241] showed that both risperidone and haloperidol reduce superoxide dismutase levels in schizophrenia patients, even though they do not normalize initially increased levels of plasma nitric oxide levels. In this study, lower superoxide dismutase (SOD) levels at baseline predicted greater symptom improvement, and greater change in SOD levels correlated with greater symptom improvement.

Paroxonase 1 (PON1) is an antioxidant enzyme synthesized in the liver which protects high density lipoprotein against oxidative stress damage. In a study by Noto et al., drug naïve FEP patients showed decreased PON1 activity and increased total radical trapping antioxidant parameter (TRAP) values [242]. TRAP levels are determined by the effects of specific non-enzymatic antioxidants in the plasma such as uric acid, albumin, bilirubin, protein-bound SH (thiol) groups, and vitamin E and C [243]. Treatment with risperidone increased PON1 and decreased lipid hydroperoxides (LOOH) levels, suggesting that risperidone may modulate oxidative stress pathways possibly through anti-oxidative activities by increasing PON1 activity and lowering a marker of lipid peroxidation (LOOH). Also, a study of the oxidative stress in first episode psychosis showed that the total level of peroxides was higher in these patients than in normal controls, and that difference resolved with anti-psychotic treatment [244].

#### Predictors of response to anti-psychotics

The ability to predict if a patient will respond to a particular treatment by the use of quantifiable biomarkers would allow individualized treatment options. This would enable the selection of treatments that are more likely to succeed for a particular patient, avoiding multiple and unnecessary trials, which can lead to patient non-adherence and relapse. In Table 6, we summarize existing evidence from studies of predictors of response to anti-psychotic treatment.

**Table 6:** Predictors of response to antipsychotics

Biomarker	Response to antipsychotic	ASD	Schizophrenia
Genes			
*HTR2A* c.-1438G>A, *DRD3* Ser9Gly, *HTR2C* c.-995G>A and *ABCB1* c.1236C>T polymorphisms	Polymorphisms that predicted clinical improvement with risperidone in children with ASD	Correia et al., 2010	NF
GBP6, RABL5, RNF213, NFKBID, and RNF40	Correlated with response to risperidone	Lit et al., 2012	NF
Multiple genes associated with neuronal cell growth	Associated with a positive response to risperidone	Bent & Hendren, 2010	NF
A-2518G polymorphism of the MCP-1	Treatment resistance associated with the G-allele	NF	Mundo et al., 2005
SNAP-25 (Mnll polymorphism)	Associated with changes in PANSS after 14 weeks of antipsychotics	NF	Muller et al., 2005
5HTT gene	Association between the 5-HTT-LPR variants and early negative symptom response to treatment in FEP	NF	Vazquez-Bourgon et al., 2010
Minerals			
↓ body zinc status (while taking risperidone)	Associated with greater improvement in irritability	Arnold et al., 2010	NF
Hormones			
↓insulin levels at T0	Predictors of symptom improvement after antipsychotic treatment	NF	Schwarz, Guest, 2012
leptin, proinsulin, TGF-α	Associated with differences between the short- and long-term relapse		
↑leptin, insulin, C-peptide	In patients who relapsed later but no change in those relapsing earlier		
Blunted CAR Diurnal cortisol levels CAR	FEP compared to controls negatively correlated with the number of recent stressful events positively correlated with a history of sexual childhood abuse	NF	Mondelli et al., 2010
Persistent ↓CAR, ↑IL-6 and IFN-ϒ	In non-responders (12 week treatment with antipsychotics) in FEP	NF	Mondelli et al., 2015
More blunted CAR	Associated with worse cognitive function	NF	Aas et al., 2011
Baseline postdexamethasone cortisol levels Persistent non-suppression of cortisol levels following the dexamethasone test after 4 weeks of antipsychotics	Unrelated to outcome at 4 weeks or 1 year Associated with poor clinical outcome	NF	Tandon et al., 1991
Neurotransmitters and metabolites			
↓pMHPG (plasma 3-methoxy-4-hydroxyphenylglycol)	FEP patients who responded to treatment (8 weeks of antipsychotics)	NF	Nagaoka et al., 1997
↑baseline pHVA and week-1 pHVA (plasma homovanillic acid) levels	In responders compared to non-responders of FEP following 6 weeks of antipsychotic treatment	NF	Koreen et al., 1994
Relatively normal striatal dopamine synthesis and elevated anterior cingulate cortex glutamate levels	Treatment resistance in schizophrenia	NF	Demjaha et al., 2014
↑pretreatment prolactin response to D-fenfluramine test	↓ response to haloperidol in FEP	NF	Mohr et al., 1998
3-OHKY (3-hydroxykynurenine) quinolinic acid at baseline	Predicted improvement following 4 week treatment with antipsychotics in FEP (lowest concentrations associated with the greatest improvement)	NF	Condray et al., 2011

*NF* no studies found

The contribution of genes in schizophrenia and ASD has been the focus of several studies which have been subject of other reviews [245, 246]. However, the role of gene expression in predicting treatment response has been less studied so far Correia et al. [247] explored the effects of multiple candidate genes on clinical improvement and occurrence of adverse drug reactions with risperidone, in children with ASD. The study included genes involved in the pharmacokinetics (*CYP2D6* and *ABCB1*) and pharmacodynamics (*HTR2A*, *HTR2C*, *DRD2*, *DRD3*, and *HTR6*) of the risperidone, and the *BDNF* gene. Results showed that the *HTR2A* c.-1438G > A, *DRD3* Ser9Gly, *HTR2C* c.-995G > A, and *ABCB1* c.1236C > T polymorphisms were predictors of clinical improvement with risperidone therapy. Likewise, Lit et al. found that expression of a group of five genes (GBP6, RABL5, RNF213, NFKBID, and RNF40) prior to treatment initiation was correlated with response to risperidone [248]. Interestingly, RNF40 is located at 16p11.2, a region implicated in both ASD and schizophrenia, and RNF40 and RNF213 have RING domains (Really Interesting New Gene domains (RING)) which contain zinc binding sites. In a study by Arnold et al. [249], in children with autistic disorder, decrease in body zinc status while taking risperidone was strongly associated with greater improvement in irritability. Another study [14] refers that multiple genes associated with neuronal cell growth are associated with a positive response to risperidone in children with ASD.

Several studies have explored polymorphisms of genes coding for dopamine and serotonin receptors of anti-psychotic drugs and treatment response in patients with schizophrenia. Signal transduction genes have also been explored, as have cytochrome polymorphisms, and polymorphisms associated with side effects. A significant association was found between the A-2518G polymorphism of the CCL2 gene and treatment resistance, with resistant patients more frequently carrying the G-allele [250].

The SNAP-25 is a synaptosomal-associated protein directly involved in the release of neurotransmitters. Muller et al. [251] studied patients with schizophrenia or schizoaffective disorder, with prior suboptimal response to anti-psychotic treatment, and found that patients with the SNAP-25 gene variant (Mnll polymorphism) had significant changes in PANSS scores after a 14-week treatment with anti-psychotics. In another study [252] with drug naïve FEP patients, the authors found significant associations between 5-HTT-LPR variants and early negative symptom response to treatment. Genetic variations in the 5-HTT-LPR polymorphism had previously been associated with variations in anti-psychotic drug response [253–255] but mainly to clozapine in samples of chronic patients [252]. In summary, so far, there is no predictor as to which patient (with a first psychotic episode) will respond to which treatment based on genetic biomarkers [9]. In a study with schizophrenia patients who were initially unmedicated or anti-psychotic naïve [256], molecular signatures that could predict symptom improvement over the first 6 weeks of treatment were described. Lower insulin levels at T0 were predictive of symptom improvement after anti-psychotic treatment, and the three molecules with the greatest differences between the short- and long-term relapse groups were leptin, proinsulin, and TGF-α. Further, leptin, insulin, and C-peptide increased significantly in patients who relapsed later but showed no change in the group that relapsed earlier.

The function of hypothalamic–pituitary–adrenal (HPA) axis in schizophrenia patients has been extensively reviewed by Bradley and Dinan [257]. Even though the authors conclude that there seems to be clinically relevant HPA axis dysfunction in patients with schizophrenia, results are heterogenous across studies and should be interpreted taking into account possible confounders. There seems to be evidence of elevated basal cortisol in some but not all patients compared to controls. However, basal cortisol secretion is influenced by psychological stress which undermines a fair comparison between patients and controls, in that psychological stress derived from the illness itself (and the presence of psychotic symptoms) and hospitalization by mental illness is irreproducible. Also, anti-psychotic drugs can influence cortisol secretion in several ways, either by reducing psychotic symptoms (and reducing the psychological stress associated with them) or by a more direct result of their pharmacological action.

Some studies have investigated the function of the HPA axis in predicting response to anti-psychotic treatment. Mondelli et al. 2010, in a study with FEP patients, reported that anti-psychotics normalized diurnal cortisol hypersecretion but failed to normalize the blunted CAR [258]. In a later longitudinal study with FEP patients [259], non-responders to 12-week treatment with anti-psychotics showed persistently lower CAR and higher IL-6 and IFN-ϒ levels when compared to responders. Blunted CAR and the reduced HPA axis reactivity to stress have also been associated with more severe symptoms and worse cognitive function in patients with psychosis [260]. Further, more blunted CAR but not more elevated cortisol levels during the day appears to be associated with cognitive dysfunction in these patients [260]. Persistent non-suppression of cortisol levels following the dexamethasone test after 4 weeks of anti-psychotic treatment has also been associated with poor clinical outcome [261].

Taking into consideration that the therapeutic effect of anti-psychotic drugs is attributed to their capacity to block dopamine and serotonin receptors, it seems likely that neurotransmitter metabolites should be investigated as potential predictors of treatment response. Two studies in the 90s [262, 263] reported elevated levels of dopamine (plasma homovanillic acid and pHVA) and decreased levels of noradrenalin (plasma 3-methoxy-4-hydroxyphenylglycol and pMHPG, respectively) metabolites in the plasma of patients with FEP who responded to treatment with anti-psychotics. A more recent study [264] found that treatment resistance in schizophrenia was associated with relatively normal striatal dopamine synthesis but elevated glutamate levels in the anterior cingulate cortex [264]. Another study [265] found that increased pretreatment prolactin response to the D-fenfluramine test, an indirect measure of serotonin activity, was associated with non-response to haloperidol in FEP patients.

More recently, Condray et al. [266] found that levels of 3-hydroxykynurenine (3-OHKY) quinolinic acid, a product of tryptophan metabolism (which is neurotoxic), not only predicted severity of clinical symptoms but also the degree of clinical improvement following brief treatment with anti-psychotics in patients with first-episode psychosis. However, studies are needed to replicate these findings in larger samples and further elucidate the value of such metabolites as treatment predictors.

It is nowadays evident that patients do not respond in the same way to anti-psychotic drugs. While intervention in dopaminergic neurotransmission seems to work for many patients, it is clearly insufficient for clinical response in others. Howes et al. [267] propose a subtyping of schizophrenia based on the underlying neurobiological mechanism rather than the current phenomenological approach which would be more useful to guide diagnostic testing and treatment. In type A schizophrenia hyperdopaminergia underlies the onset of the disorder and symptoms, and these patients show good response to dopamine-blocking anti-psychotics. In contrast, type B schizophrenia patients have normal dopaminergic function and symptoms that are unrelated to dopaminergic function, with glutamatergic alterations being a probable candidate among others [267]. Further studies are needed to help clarify the underlying biological mechanism of illness and to help determine which patients would benefit from which type of anti-psychotic treatment.

### Does gender matter?

Males are four to eight times more likely than females to have a developmental disorder, such as ASD. Recent advances in neuroscience suggest explanations as to why the male gender is a vulnerability factor. Brain development in males is affected by androgen production in fetal testis, leading to differences in neuroanatomy and physiology, relative to female brain. These developmental differences have been recently reviewed [268]. Overall, females appear to be able to withstand a greater amount of insult, and thus pre-natal insult likely impacts males more than females. Also, the male brain has been suggested to be less able to compensate once damage occurs. Several aspects of developmental sex differences and their consequences for health and disease have been the object of reviews [58, 94, 268, 269].

The involvement of the placenta in fetal growth and development is also being considered. As the trophoblast cells originate from the embryo they can be XX or XY, and that may condition placental biochemistry and function. The work of Tracy Bale on a pre-natal stress animal model where male offspring showed several altered behaviors, showed alterations in male but not in female placental gene expression, when compared to controls [270–272]. In a previous study, they found differences in male placental expression of genes related to growth and development, but no significant differences in inflammatory cytokines measured, like TNF-α [270]. However, in a more recent work, they showed that pro-apoptotic factor FasL was affected in placentas of both sexes, while other important inflammatory mediators, like IL-6 or CCR7, were overexpressed in male but not female placentas after maternal stress [272]. Importantly, the expression of these mediators reverted to normal when dams were treated with anti-inflammatory molecules (NSAIDS) [272]. These and other aspects of sex-specific transplacental signals to the developing brain were recently reviewed [271].

It has been suggested that males and females may have fundamentally different response to neonatal activation of the immune system [94], which may be linked to the differences in response to external stimuli, such as LPS during early post-natal period. LPS induces depressed activity of peripheral immune cells in adult rat males but not in adult rat females. The mechanism suggested involves enzymatic cleavage by caspase-1 of the pro-form of IL1-β into its active form, the female brain being more protected to this process when it occurs early in life [94]. On the other hand, microglia and inflammatory signaling molecules, particularly PGE2 pathway, play major roles in male brain sexual differentiation [267]. Importantly, PGE2 levels were reported as increased in plasma of male ASD patients compared to controls [273], and in a previous study also increased in the plasma of schizophrenia patients [274].

Sex-specific differences in potential biomarkers have been reported in ASD, highlighting the importance of stratification by sex. Steeb et al. [275] showed that adult women with Asperger’s syndrome (AS) had alterations in proteins mostly involved in lipid transport and metabolism pathways, while men with AS showed changes predominantly in inflammation signaling. Another study found that in men with ASD the predominant biomarker signature was increased levels of cytokines and inflammatory molecules, contrasting with altered levels of growth factors and hormones in women patients [276].

In schizophrenia studies, stratification by sex is also important. Ramsey et al. [277] compared first-episode anti-psychotic naïve schizophrenia patients and controls to identify sex-specific differences in peripheral blood biomarkers. In female patients, the inflammation-related molecules alpha-1-antitrypsin, B lymphocyte chemoattractant BLC and IL-15 showed negative associations with positive and total PANSS, positive PANSS, and negative and total PANSS, respectively. In male patients, the hormones prolactin and testosterone were negatively associated with positive PANSS ratings. In the same study [277], the authors investigated molecular changes in a subset of 33 patients before and after 6 weeks of treatment with anti-psychotics. Results showed that treatment induced sex-specific changes in the levels of testosterone, serum glutamic oxaloacetic transaminase, follicle-stimulating hormone, IL-13, and macrophage-derived chemokine, highlighting the fact that anti-psychotics may have different effects in men and women.

Further advances into the biological origins of male vulnerability and female protection will undeniably contribute to novel targets for therapeutic intervention and prevention.

## Conclusions

Schizophrenia and ASD are complex disorders which have been classically seen and investigated as separate illnesses. In this review, we have gathered research that bridges ASD and schizophrenia through inflammation and biomarkers, spanning from pre-clinical animal model studies to clinical research.

The link between inflammation and the development of ASD or schizophrenia can provide new explanations for the occurrence of these disorders and can lead to the identification of quantifiable biological biomarkers, which would be invaluable in early diagnosis and treatment. Different classes of biomarkers have been investigated, including at the clinical level with patient cohorts. Heterogeneity of patient conditions within and across different cohorts, and the small numbers of individuals that end up being included in the study, are some of the difficulties still faced by these studies. Although it is now generally accepted that pro-inflammatory mediators, such as cytokines, are increased in the plasma of ASD and schizophrenia patients, so far there is not a validated panel that can provide a reliable inflammatory signature for these disorders. So, further validation of these potential biomarkers is still required. Moreover, recent research on miRNAs expression pattern as biomarkers is very promising, as these molecules could provide not just biomarkers, but new therapeutic approaches to correcting cellular processes that may be unbalanced. Moreover, particularly in clinical research, where patient cohorts will likely be medicated, the effect of medication and the impact of gender need always to be considered when designing new studies and interpreting the results obtained. In this context, the work in animal models is essential, but is still concentrated in establishing their validity at different levels. Interestingly, some animal models show good validity for both schizophrenia and ASD, particularly those focused on MIA, supporting that inflammation may be an important common link between these disorders. Moreover, evidence of their good performance in response to treatment with anti-psychotics is a good indicator of their potential to be used in biomarker discovery. On the other hand, given the known differences both at the neuropsychiatric level and the immune system level, any biomarkers studied in animal models will require careful validation in clinical studies. Both in pre-clinical and clinical studies, the role of microglia and the key molecular pathways that activate these cells are of crucial importance. Microglia response can shed light not only on the local production of pro-inflammatory mediators in the brain but also on the link between systemic and local inflammation and the mechanisms involved.

In conclusion, this is an exciting area of research, where state-of-the-art molecular tools and the use of animal models will greatly contribute to uncovering new biomarkers. These will likely be related to inflammatory processes, and require a panel of molecules rather than one single molecule, in order to improve their sensitivity and specificity. Validating those biomarkers for use in clinical practice could markedly improve diagnostics, patient stratification and treatment monitoring, improving patient care for these that are still debilitating chronic illnesses.

## Abbreviations

APLAs: Anti-phospholipid antibodies
AS: Asperger’s syndrome
ASD: Autism spectrum disorder
BDNF: Brain-derived neurotrophic factor
BH4: Biopterin
BLC: B lymphocyte chemoattractant
CARS: Child Autism Rating Scale
CCL: Chemokine (C-C motif) ligand
CD: Cluster of differentiation
CNS: Central nervous system
COX: Cyclooxygenase
CXCL: Chemokine (C-X-C motif) ligand
DISC1: Disrupted in schizophrenia
DN: Dominant-negative
ERK: Extracellular signal-regulated kinase
GFAP: Glial fibrillary acidic protein
GM-CSF: Granulocyte macrophage-colony stimulating factor
GRIA2: Glutamate ionotropic receptor AMPA type subunit 2
GSH: Glutathione
GSK: Glycogen synthase kinase
HIV: Human immunodefficiency virus
HLA-DR: Human leukocyte antigen-antigen D related
HPA: Hypothalamic–pituitary–adrenal
Ig: Immunoglobulin
IL: Interleukin
INF: Interferon
i-NOS: Inducible nitric oxide (NO)-synthase
KO: Knock out
LI: Latent inhibition
LPS: Lipopolysaccharides
MeCP2: Methyl-CpG-binding protein 2
mGluR5: Metabotropic glutamate receptor type 5
MHC: Major histocompatibility complex
MIA: Maternal immune activation
miRNA: microRNAs
MMPs: Matrix metalloproteinases
MPEP: Methyl-6-(phenylethynyl)-pyridine
MPP: Mouse Phenome Project
MRI: Magnetic resonance imaging
m-RNA: messenger RNA
NF-**k**B: Nuclear factor kappa-light-chain enhancer of activated B cells
NK: Natural Killer
NMDA receptor: *N*-methyl-D-aspartate receptor
NRG1: Neuregulin 1
OPN: Osteopontin
PANSS: Positive and negative syndrome scale
PCP: Phencyclidine
PET: Positron emission tomography
Poli (I:C): Polyriboinosinic-polyribocytidilic acid
PON1: Paroxonase 1
PPI: Pre-pulse inhibition
PTEN: Phosphatase and tensin homolog
PV: Parvalbumin
RING: Really interesting new gene
ROR**γ**t: Retinoic acid receptor-related orphan nuclear receptor **γ**t
SERPINA1: Serpin family A member 1
Shank3: SH3 and multiple ankyrin repeat domains 3
SOD: Superoxide dismutase
TGF: Transforming growth factor
Th: T Helper
TLR: Toll-like receptor
TNF: Tumor necrosis factor
TRAP: Trapping antioxidant parameter
TREM2: Triggering receptor expressed on myeloid cells 2
TSPO: Mitochondrial translocator protein 18 kDa
TYROBP: TYRO protein tyrosine kinase-binding protein
USV: Ultrasonic vocalization
VPA: Valproic acid
